# Transcriptional Regulation of Rod Photoreceptor Homeostasis Revealed by *In Vivo* NRL Targetome Analysis

**DOI:** 10.1371/journal.pgen.1002649

**Published:** 2012-04-12

**Authors:** Hong Hao, Douglas S. Kim, Bernward Klocke, Kory R. Johnson, Kairong Cui, Norimoto Gotoh, Chongzhi Zang, Janina Gregorski, Linn Gieser, Weiqun Peng, Yang Fann, Martin Seifert, Keji Zhao, Anand Swaroop

**Affiliations:** 1 Neurobiology-Neurodegeneration and Repair Laboratory, National Eye Institute, National Institutes of Health, Bethesda, Maryland, United States of America; 2 Genomatix GmbH, Munich, Germany; 3 Information Technology and Bioinformatics Program, National Institute of Neurological Disorders and Stroke, National Institutes of Health, Bethesda, Maryland, United States of America; 4 Laboratory of Molecular Immunology, National Heart, Lung, and Blood Institute, National Institutes of Health, Bethesda, Maryland, United States of America; 5 Department of Physics, The George Washington University, Washington, D.C., United States of America; Stanford University School of Medicine, United States of America

## Abstract

A stringent control of homeostasis is critical for functional maintenance and survival of neurons. In the mammalian retina, the basic motif leucine zipper transcription factor NRL determines rod versus cone photoreceptor cell fate and activates the expression of many rod-specific genes. Here, we report an integrated analysis of NRL-centered gene regulatory network by coupling chromatin immunoprecipitation followed by high-throughput sequencing (ChIP–Seq) data from Illumina and ABI platforms with global expression profiling and *in vivo* knockdown studies. We identified approximately 300 direct NRL target genes. Of these, 22 NRL targets are associated with human retinal dystrophies, whereas 95 mapped to regions of as yet uncloned retinal disease loci. *In silico* analysis of NRL ChIP–Seq peak sequences revealed an enrichment of distinct sets of transcription factor binding sites. Specifically, we discovered that genes involved in photoreceptor function include binding sites for both NRL and homeodomain protein CRX. Evaluation of 26 ChIP–Seq regions validated their enhancer functions in reporter assays. *In vivo* knockdown of 16 NRL target genes resulted in death or abnormal morphology of rod photoreceptors, suggesting their importance in maintaining retinal function. We also identified histone demethylase Kdm5b as a novel secondary node in NRL transcriptional hierarchy. Exon array analysis of flow-sorted photoreceptors in which *Kdm5b* was knocked down by shRNA indicated its role in regulating rod-expressed genes. Our studies identify candidate genes for retinal dystrophies, define *cis*-regulatory module(s) for photoreceptor-expressed genes and provide a framework for decoding transcriptional regulatory networks that dictate rod homeostasis.

## Author Summary

The rod and cone photoreceptors in the retina are highly specialized neurons that capture photons under dim and bright light, respectively. Loss of rod photoreceptors is an early clinical manifestation in most retinal neurodegenerative diseases that eventually result in cone cell death and blindness. The transcription factor NRL is a key regulator of rod photoreceptor cell fate and gene expression. Here, we report an integrated analysis of the global transcriptional targets of NRL. We have discovered that both NRL and CRX binding sites are present in genes involved in photoreceptor function, implying their close synergistic relationship. *In vivo* loss-of-function analysis of 16 NRL target genes in the mouse retina resulted in death or abnormal morphology of photoreceptor cells. Furthermore, we identified histone demethylase Kdm5b as a secondary node in the NRL-centered gene regulatory network. Our studies identify NRL target genes as excellent candidates for mutation screening of patients with retinal degenerative diseases, and they provide the foundation for elucidating regulation of rod homeostasis and targets for therapeutic intervention in diseases involving photoreceptor dysfunction.

## Introduction

Molecular mechanisms underlying neuronal differentiation and generation of complex sensory and behavioral circuits in the mammalian central nervous system are still poorly elucidated. Gene regulatory networks (GRNs) integrate key control elements that guide the development of distinct cell types [1], [2], [3] and contribute to precise maintenance of diverse cellular functions. As perturbations in homeostatic mechanisms (e.g., during aging and disease) can cause dysfunction or death of neurons [4], [5], a better understanding of GRNs that control neuronal homeostasis would augment the design of therapies for neurodegenerative diseases.

The rod and cone photoreceptors in mammalian retina are highly specialized neurons that transduce visual signals under dim and bright light conditions, respectively [6]. Daily renewal of almost 10% of outer segment membrane discs creates high metabolic demands, making the photoreceptors vulnerable to genetic and environmental insults [7]. Rods constitute over 95% of all photoreceptors in most mammals, including mice and humans; however, cones mediate high acuity and color vision [8]. Notably, functional impairment or loss of rod photoreceptors is an early clinical manifestation in most retinal neurodegenerative diseases that eventually results in cone cell death and blindness [9], [10], [11]. The GRNs that dictate homeostatic responses in mature rod photoreceptors have not been elucidated.

During development, rod and cone photoreceptors are produced from common pools of retinal progenitors under the control of multiple transcription factors and regulatory signaling pathways [11], [12], [13]. Furthermore, the basic motif-leucine zipper protein NRL is the dominant transcription factor that determines rod photoreceptor cell fate. In *Nrl^−/−^* mice, all post-mitotic cells originally fated to become rods instead generate a cone-only photoreceptor layer [14], whereas ectopic *Nrl* expression in photoreceptor precursors produces a rod-only retina [15]. Interestingly, knock-in mice where *Nrl* is replaced by thyroid hormone receptor β2 (*Trb2*) have an M-cone dominant retina, but the presence of both NRL and TRb2 yields a normal contingent of rods [16]. A key transcriptional target of NRL is the orphan nuclear receptor NR2E3 that primarily represses cone genes to establish rod identity [17], . The cone-rod homeobox CRX is another essential transcriptional activator of photoreceptor-specific genes as rods and cones in *Crx^−/−^* mice do not develop outer segments and eventually die [20], [21], [22]. NRL and CRX continue to be expressed at high levels in mature retina and in rod photoreceptors ([23]; Gotoh, Swaroop et al. unpublished data). Protein interaction and transcriptional activation assays, combined with expression profiling of knockout mice, demonstrate that NRL and CRX are the two major regulators of rod photoreceptor gene expression [24], [25], [26], [27].

We hypothesize that detailed mapping of a rod-specific GRN would lead to the development of better therapeutic interventions in blinding diseases involving photoreceptor degeneration. Here we report the genomewide NRL *in vivo* occupancy in adult mouse retina by chromatin immunoprecipitation followed by high-throughput sequencing (ChIP–Seq) using Illumina and ABI sequencing platforms. We perform an integrated analysis by coupling the NRL ChIP–Seq data with published photoreceptor-specific transcriptional profiles and CRX ChIP–Seq results. We use *in vivo* knockdown assays to examine the physiological relevance of NRL target genes and identify secondary regulatory nodes downstream of NRL in rod transcriptional hierarchy. Our studies establish NRL and CRX as the key regulatory nodes for rod-expressed genes, identify NRL targets as candidate genes for retinal diseases, and provide a framework for GRN that controls homeostasis in rod photoreceptors.

## Results

### Genome-Wide Mapping of NRL Occupancy by ChIP–Seq

**Figure 1:**
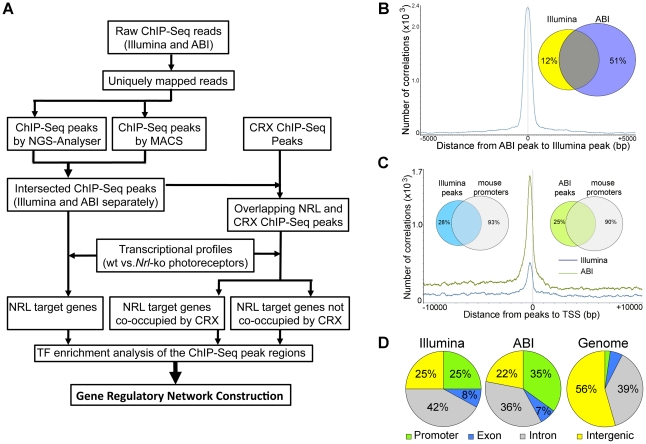
Genome-wide Occupancy of NRL revealed by ChIP–Seq using Illumina and ABI/SOLiD sequencing platforms. (A) Analysis workflow. Raw sequence reads from Illumina or ABI/SOLiD were mapped to the mouse genome (NCBI build 37) using the Genomatix Mining Station (GMS) and the reads mapped to unique genomic locations (uniquely mapped reads) were used for further analyses. ChIP–Seq peaks were called using NGS Analyzer (Genomatix) or MACS (Zhang et al., 2008), and the common peaks were used for further analyses. The NRL ChIP–Seq peaks were compared to the CRX ChIP–Seq peaks for overlapping using GenomeInspector (Genomatix) software. The ChIP–Seq peaks were assigned to the nearest gene. Transcription profile analyses of flow-sorted photoreceptors of WT and *Nrl* ^−/−^ were performed using ChipInsepector program (Genomatix) and 1.5 fold expression change was used as a criterion for NRL target genes. TF motif enrichment analyses were performed on the NRL ChIP–Seq peak regions that were associated with NRL target genes. Comparison was made between CRX-overlapping and non CRX-overlapping NRL ChIP–Seq peaks. Gene regulatory network was constructed based on TF enrichment analysis. (B) Correlation of ChIP–Seq peaks by Illumina and ABI. The number of correlations (y-axis) was plotted to the distance of ABI ChIP–Seq peaks to Illumina ChIP–Seq peaks (x-axis). The Venn diagram (inset) calculated the percentage of ABI and Illumina peaks within 500 bp of each other: 88% of Illumina peaks are within 500 bp of ABI peaks and 49% of ABI peaks are within 500 bp of Illumina peaks. (C) Correlation of ChIP–Seq peaks to promoters. The number of correlation (y-axis) was plotted to the distance of ABI ChIP–Seq peaks (green graph) or Illumina ChIP–Seq peaks (blue graph) to the transcription start site (TSS) (x-axis). The Venn diagram (inset) calculated the percentage of ABI (75%) or Illumina (72%) peaks within 10,000 bp from the TSS. (D) Genomic distribution of NRL ChIP–Seq peaks relative to the nearest annotated genes. Promoters and exons account for 2.3% and 5.4% of the mouse genome, respectively.

We performed chromatin immunoprecipitation experiments using anti-NRL antibody (with normal IgG as a control) to pull down the genomic fragments bound by NRL *in vivo* in adult mouse retina. The ChIP DNA was subjected to direct high-throughput sequencing using either Illumina 1G genome analyzer or ABI/SOLiD system (ABI). The workflow for the analysis of two datasets is shown in Figure 1A. (see www.nei.nih.gov/intramural/nnrldataresource.asp for raw sequence reads). Illumina and ABI datasets contained a total of 8 million 25-bp reads and 18.0 million 35-bp reads, respectively. Of these, respectively 5.3 million (66.3%) and 6.3 million (35%) reads were uniquely mapped to the mouse genome (NCBI Build 37, UCSC mm9), with overlapping sequence reads forming the NRL ChIP–Seq peaks (Table 1). We used NGS Analyzer (Genomatix) and MACS [28], in parallel, to determine NRL ChIP–Seq peaks with ChIP–Seq counts from negative control (normal IgG) libraries as thresholds. The peaks identified by both algorithms (intersected peaks) were kept for further analyses (Figure 1A, Table 2). Illumina and ABI platforms revealed 2790 and 5625 NRL ChIP–Seq peaks, respectively (Table 2). The number of peaks did not correlate with chromosome size (data not shown), indicating the interaction of NRL with specific genomic regions. The average peak widths were 398 bp (Illumina) and 408 bp (ABI), with average peak heights being 58.0 (Illumina) and 79.9 (ABI) and median peak heights of 30 (Illumina) and 47 (ABI) (Table 2). Illumina and ABI ChIP–Seq peak centers showed a strong correlation (Figure 1B), with almost 90% of Illumina peaks overlapping with ABI peaks (Figure 1B).

**Table 1:** Comparison of ChIP–Seq peaks by Illumina and ABI.

Reads	Illumina		ABI	
	NRL Ab	IgG control	NRL Ab	IgG control
Total sequenced reads (millions)	8	8.3	18	13.8
Uniquely mapped reads (millions)	5.3	5.1	6.3	3.7
Percent of uniquely mapped reads	66.3	61.4	35	27.1

ChIP–Seq libraries were prepared according to manufacture's instructions and sequenced by Illumina 1G Genome Analyzer or ABI/SOLiD platform. Uniquely mapped reads: reads mapped to unique genomic locations.

**Table 2:** Comparison of ChIP–Seq peaks by Illumina and ABI.

Peaks	Illumina			ABI		
	MACS	NGS	Common	MACS	NGS	Common
						
Peak number	5006	2968	2790	8168	12326	5625
Peak coverage (×10^6^ bp)	1.8	0.7	1.1	3	2.4	2.3
Average width (bp)	350	245	398	366	193	408
Average height	24.2	57.7	58	26	48	79.9
Median height	15	27	30	16	23	47
Minimum height	6	11	11	6	15	15

ChIP–Seq peaks were identified using MACS with p<10^−6^ or NGS-Analyzer (Genomatix) with p<10^−3^.

A large number of NRL ChIP–Seq peaks were mapped within 1 kb of the transcription start sites (Figure 1C, Table S1). Furthermore, over 70% of NRL ChIP–Seq peaks were present within 10 kb of 7–10% of mouse gene promoters (see Venn diagram in Figure 1C). The NRL ChIP–Seq peaks from both Illumina and ABI platforms are highly enriched in promoter regions, given that promoters only account for approximately 2% of the mouse genome (Figure 1D).

### Integration of ChIP–Seq with Expression Profiling

**Figure 2:**
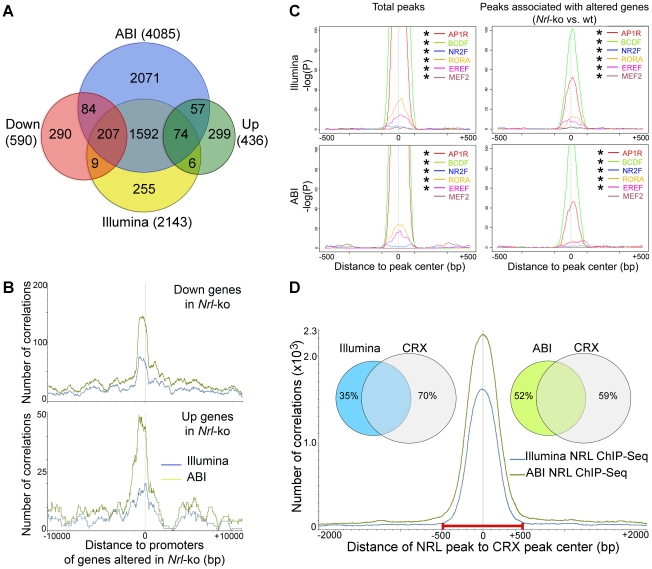
Identification of NRL target genes and co-regulatory transcription factors by ChIP–Seq and transcriptional profiling. (A) Identification of direct NRL transcriptional target genes by ChIP–Seq and transcription profiling. ABI ChIP–Seq peaks and Illumina ChIP–Seq peaks were assigned to the nearest genes (ABI and Illumina). Transcriptional profiling of flow-sorted photoreceptors of WT or *Nrl* ^−/−^ mice was generated using microarrays. Up: genes up-regulated in *Nrl* ^−/−^ photoreceptors. Down: genes down-regulated in *Nrl* ^−/−^ photoreceptors. (B) Correlation of NRL ChIP–Seq peaks to the promoters of its target genes. The number of correlation (y-axis) was plotted to the distance (x-axis) of ABI ChIP–Seq peaks (green graph) or Illumina ChIP–Seq peaks (blue graph) to the promoters of the genes that were down-regulated (top) or up-regulated (bottom) in the *Nrl* ^−/−^ photoreceptors. (C) TF enrichment and TF binding site (TFBS) positional bias analysis. NRL ChIP–Seq peak regions were analyzed for TF enrichment using Genomatix RegionMiner. The positional bias of TFBS (P) was calculated and plotted as −log(P) (y-axis) to the distance of TFBS to the peak center (x-axis). Positions where TFBS are overrepresented appear as peaks in these plots. * significantly enriched. (D) Correlation of NRL ChIP–Seq peaks with CRX ChIP–Seq peaks. The number of correlation (y-axis) was plotted to the distance of NRL ChIP–Seq peaks to CRX ChIP–Seq peaks (x-axis). The Venn diagram (inset) calculated the percentage of NRL ChIP–Seq peaks (Illumina and ABI) within 500 bp of CRX peaks: 65% of Illumina peaks and 48% of ABI peaks are within 500 bp of CRX peaks.

In order to identify physiologically relevant NRL target genes, we examined Illumina and ABI ChIP–Seq data in combination with global expression profiles of flow-sorted photoreceptors from wild type (WT) and *Nrl* ^−/−^ mouse retina [25]. Of 2143 genes associated with Illumina ChIP–Seq peaks, 216 exhibited at least 1.5 fold less expression and 80 genes showed higher expression in *Nrl^−/−^* photoreceptors (Figure 2A). Of 4085 genes associated with ABI ChIP–Seq data, we identified 291 genes with lower and 131 genes with higher expression in the *Nrl^−/−^* photoreceptors (Figure 2A). A combined analysis of Illumina and ABI ChIP–Seq datasets yielded 281 genes showing altered expression in *Nrl^−/−^* photoreceptors. A high correlation was detected between NRL ChIP–Seq peaks (from both Illumina and ABI datasets) and promoters of genes that are differentially expressed in rod photoreceptors of WT *versus Nrl^−/−^* retina (Figure 2B). For convenience, we will refer genes associated with NRL ChIP–Seq peaks and altered in *Nrl^−/−^* retina as direct transcriptional targets of NRL.

### Enrichment of Co-Regulatory Modules within NRL ChIP–Seq Peaks

As transcription factor interactions determine the specificity of gene expression patterns [29], [30], we performed motif enrichment analysis (Genomatix RegionMiner, “Over-represented transcription factor binding sites” based on MatInspector [31], [32]) of sequences under the NRL ChIP–Seq peaks. As predicted, we noticed a significant enrichment of the binding sites for NRL and other AP1 related factors (AP1R) in peaks associated with genes that are up- or down-regulated in the absence of *Nrl* (Table S2).

An unbiased motif enrichment analysis of ChIP–Seq peak regions for NRL targets revealed binding sites for transcription factor families that include key photoreceptor regulatory proteins – CRX (BCDF family) [20], [24], [33], NR2E3 (NR2F family) [18], [19], [34], [35], RORβ (RORA family) [36], [37], ESRRβ (EREF family) [38] and MEF2C (MEF2 family) [39] (Table S2). Motifs for these transcription factors were significantly enriched within total NRL ChIP–Seq peaks and within the peaks associated with genes that are differentially expressed in *Nrl^−/−^* photoreceptors (except for MEF2C in ABI data) (Figure 2C). The motifs for AP1R (NRL), BCDF (CRX), RORA (RORβ) and EREF (ESRRβ) families were located close to the peak center whereas motifs for NR2E3 and MEF2C were not (Figure 2C). The composition and enrichment ranking of enriched transcription factor motifs were different between NRL target genes whose expression was down- or up-regulated in *Nrl^−/−^* photoreceptors (Table S2), suggesting that NRL cooperated with different proteins to activate or repress gene expression. However, the genomic distribution of NRL peaks is similar among the various groups (Figure S1).

As CRX is an established transcriptional activator of photoreceptor genes [21] and is shown to interact with NRL [24], we integrated NRL ChIP–Seq peaks with the previously published CRX ChIP–Seq data [22]. Interestingly, 65% of NRL ChIP–Seq peaks obtained from Illumina and 48% of those from ABI overlapped with the CRX peaks (Figure 2D), consistent with a previous finding that 51% of the down-regulated genes in *Nrl^−/−^* mice exhibit reduced expression in *Crx^−/−^* retina as well [40]. Motif enrichment analysis of NRL-CRX-overlapping peak regions and of non CRX-overlapping peaks revealed AP1R binding site (NRL) as the only common motif (Table S3). The motifs for other photoreceptor transcription factors ESRRβ, RORβ, NR2E3 were only enriched in NRL-CRX-overlapping peaks, and the most enriched motif for non CRX-overlapping NRL peaks was for SP1 family proteins (Table S3).

Ontology analysis revealed distinct biological functions for genes that were associated with NRL-CRX-overlapping ChIP–Seq peaks (involved in photoreceptor function) *versus* genes associated with non CRX-overlapping NRL peaks (basic cellular processes) (Table S4).

### Validation of *In Vivo* NRL Occupancy

**Figure 3:**
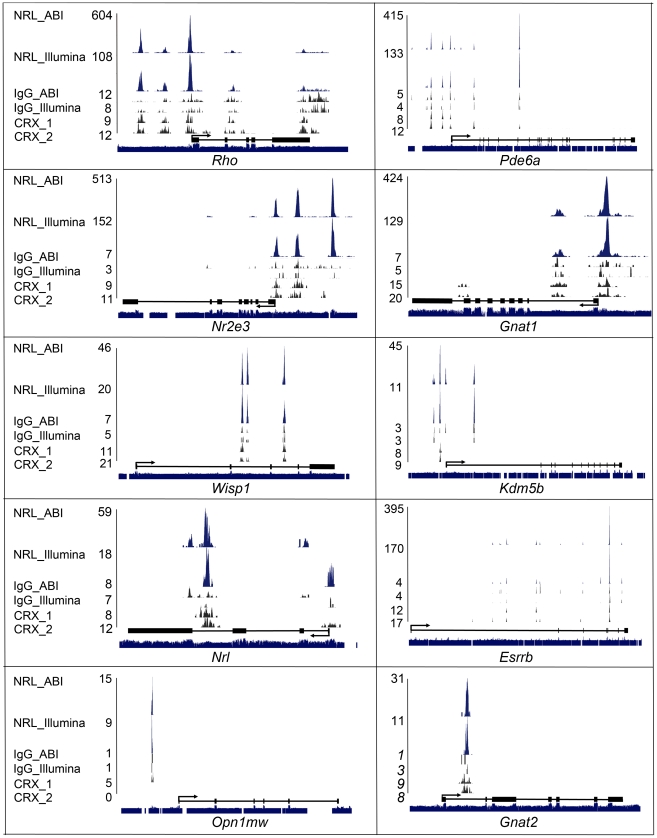
Visualization of NRL ChIP–Seq peaks and CRX ChIP–Seq peaks. NRL and CRX ChIP–Seq peaks for known and novel NRL target genes were visualized with the UCSC genome browser. NRL peaks (in blue), IgG peaks (in black) and CRX peaks (in black) represent the numbers of sequence tags detected at each location and the numbers are the peak-summit count. Exon (Black box) and intron (black line) structure are shown below peaks. Species conservation is shown at the bottom. NRL_ABI and NRL_Illumina: NRL ChIP–Seq using ABI sequencing platform and illumina sequencing platform, respectively. IgG_ABI and IgG_Illumina: IgG control ChIP–Seq using ABI sequencing platform and illumina sequencing platform, respectively. CRX_1 and CRX_2: duplicate CRX ChIP–Seq data using Illumina sequencing platform.

We first checked Illumina and ABI ChIP–Seq data for a few established NRL target genes that are involved in rod development or function (Figure 3). In addition to the reported NRL-binding sequences (at −75 bp for *Rho* and −3.5 kb for *Nr2e3*) [41], [42], [43], ChIP–Seq data further identified binding sites for NRL in *Rho* at −3 kb and −1.5 kb and in *Nr2e3* at −1 kb and −100 bp. We also detected NRL binding in rod-specific genes (such as *Pde6a*, *Gnat1*) and *Esrrb*, an important regulator of rod gene expression [38]; the expression of these genes is decreased significantly in *Nrl* ^−/−^ mice. In *Esrrb*, we identified strong NRL binding to the second intron. Interestingly, a strong NRL ChIP–Seq peak was observed within an intron of the *Nrl* gene in addition to a peak in the promoter region. *Kdm5b* and *Wisp1* are among additional genes that are regulated by NRL and play a role in rod homeostasis (see later). NRL also binds to cone-specific genes and may contribute to their down-regulation to maintain a rod phenotype, as proposed previously [11], [41].

In general, ABI ChIP–Seq peaks were higher than Illumina peaks although uniquely mapped reads in the two libraries were comparable (5.3 million *vs* 6.3 million) (Figure 3). Even though ABI data produced more peaks (e.g., *Kdm5b* and *Nrl*), Illumina data detected unique peaks that were not present in ABI (e.g., *Esrrb*) (Figure 3). We then plotted CRX ChIP–Seq peaks [22] relative to NRL peaks.

**Figure 4:**
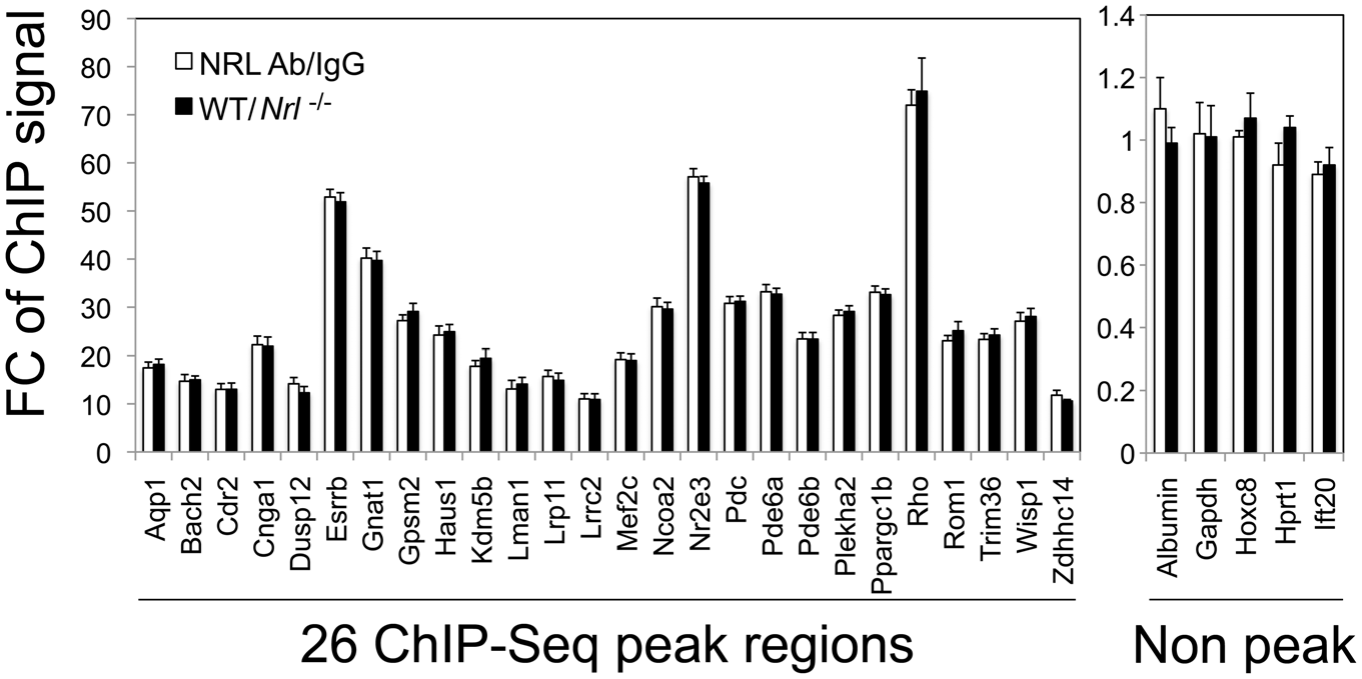
Validation of NRL binding to corresponding peak regions by ChIP–qPCR. ChIP-qPCR was performed to validate NRL binding to 26 ChIP–Seq peak regions (left panel), and 5 non-peak regions (right panel) served as negative controls. The amount of ChIP DNA was measured by qPCR in triplicates using primers flanking the regions of interest. Normal IgG served as an antibody control when ChIP was performed using WT retinas (white bars). White bars (NRL Ab/IgG) represent fold change (FC) of qPCR signals comparing NRL ChIP DNA to the IgG control ChIP DNA. A separate set of ChIP assays was performed using NRL antibody to compare signals from WT retina to signals from *Nrl* ^−/−^ retina (tissue control). Black bars (WT/*Nrl* ^−/−^) represent fold increase (Fc) of qPCR signals comparing NRL ChIP DNA from wild type C57BL/6 mouse retina to NRL ChIP DNA from *Nrl^−/^* ^−^ mouse retina. The ChIP-qPCR assays were performed twice. The representative results were shown as mean ± SD. P<0.01 for all by Student's t test.

We then performed ChIP-qPCR validations for a number of known and novel NRL targets. To strictly control the ChIP-qPCR analyses, we used two sets of controls: normal IgG as an antibody control and retina from *Nrl* ^−/−^ mice as a tissue control. We compared ChIP-qPCR signals between anti-NRL antibody and normal IgG using WT mouse retina, and performed additional NRL ChIP analysis using WT and *Nrl* ^−/−^ mouse retina (Figure 4). The two sets of experiments were highly concordant and validated the ChIP–Seq findings for all 26 sites (with various ChIP–Seq peak heights) that were tested. ChIP-qPCR analysis did not detect the association of NRL with 5 genomic regions that did not include ChIP–Seq peaks (Figure 4).

### ChIP–Seq Peak Sequences Function as Enhancer Elements

**Figure 5:**
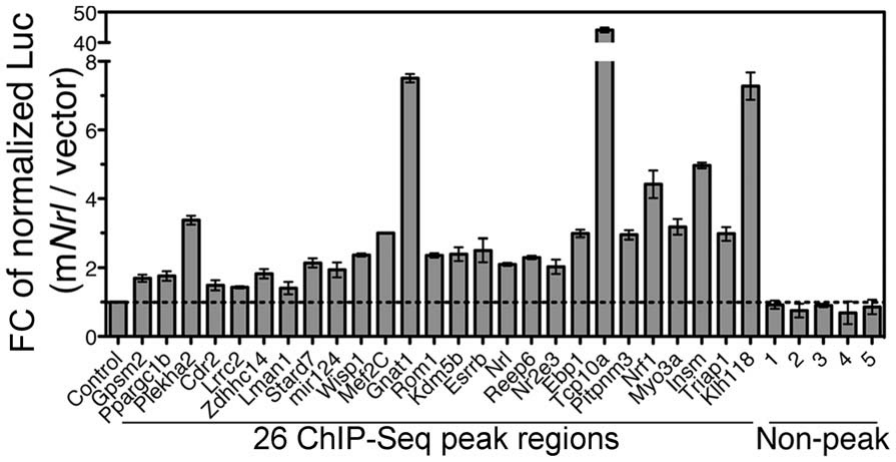
Enhancer function of NRL ChIP–Seq regions in transfected HEK293T cells. Twenty-six NRL peak regions were cloned into pGL3-promoter vector in front of a SV40 basal promoter and a luciferase reporter. The constructs were transfected in HEK293T cells together with mouse *Nrl* (m*Nrl*) expression plasmid (in pC4C vector) or empty pC4C vector. The y-axis is fold change (Fc) of normalized luciferase readings. Control: enhancer constructs co-transfected with empty pC4C vector. Five non-peak regions served as additional negative controls. The experiments were performed three times. Representative results are shown as mean ± SD. P<0.05 for all by Student's t test.

To further test the functional relevance of NRL genome occupancy detected by ChIP–Seq, we generated enhancer-reporter constructs by cloning 26 randomly chosen ChIP–Seq peak regions (with a linear range of peak tags) and 5 non-peak genomic fragments of comparable sizes upstream of an SV40 basal promoter and a luciferase reporter gene. Of 26 NRL ChIP–Seq regions, at least 19 included CRX ChIP–Seq peaks. Five non-peak genomic fragments (3′*Rho*, 3′*Pde6b*, *Gapdh*, *Hprt*, *Oct4*) were negative for CRX peaks. Co-transfection of mouse NRL expression plasmid in HEK293T cells increased the luciferase reporter expression from all 26 enhancer constructs containing NRL ChIP–Seq peaks, but not from the 5 constructs containing non-peak fragments (Figure 5). Our data suggest that the genomic fragments spanning NRL ChIP–Seq peaks can function as enhancer elements and mediate NRL-driven transcriptional activation of target genes.

We also cloned and tested NRL peak regions associated with four cone genes (*Gnat2*, *m-Opsin*, *Gngt2* and *Pik3ap1*) using the same reporter assay (2). Co-transfection of NRL expression plasmid increased the luciferase reporter expression from these enhancer constructs as well (Figure S2), validating the primary function of NRL as a transcriptional activator. However, we can not exclude the function of NRL in directly repressing cone genes *in vivo* as it may require interaction with native promoters and cis-elements, recruitment of appropriate cofactors, and/or native chromatin context, which are not provided in HEK293 cells.

### NRL Target Genes as Candidates for Retinal Diseases

We hypothesized that NRL target genes would contribute to rod photoreceptor homeostasis, and their abnormal regulation could lead to photoreceptor dysfunction and/or degeneration. We therefore integrated the chromosomal location of the human orthologs of NRL target genes with mapping information for human genetic loci for retinal diseases (RetNet http://www.sph.uth.tmc.edu/retnet/). We identified 21 NRL target genes that are known to be associated with retinal diseases involving photoreceptor degeneration (Table S5). Furthermore, almost 100 human NRL target genes map within the critical region of 29 as yet uncloned retinal disease loci (Table S5).

### *In Vivo* Functional Analysis of NRL Target Genes

**Figure 6:**
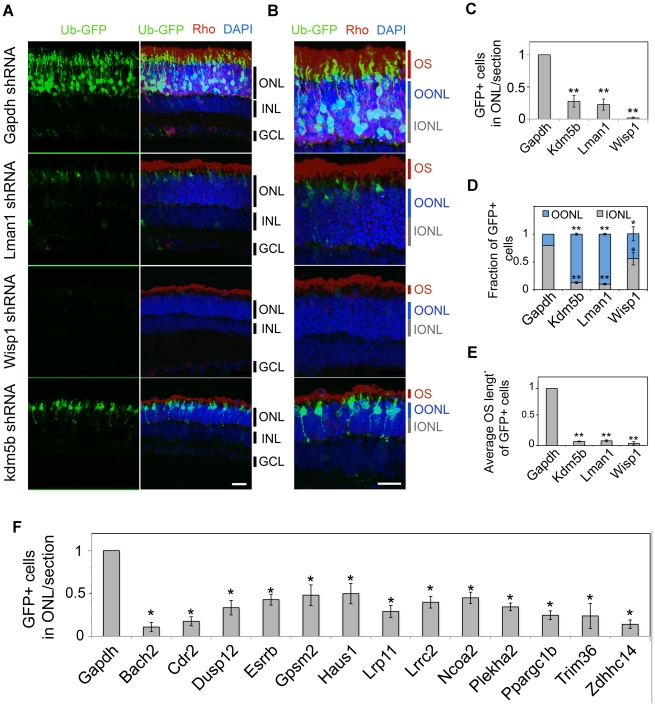
*In vivo* knockdown of NRL targets by shRNA sub-retinal injection and *in vivo* electroporation. CD-1 mouse retinas were transfected at P0 with Ub-GFP and shRNA against *Gapdh* or NRL target genes by sub-retinal injection followed by *in vivo* electroporation (A–F). Retina were harvested at P20 and examined for GFP fluorescence (green), Rho immuno-reactivity (red) and DAPI staining (blue). At least 3 biological replicate retinas were collected and imaged. (A). ONL: outer nuclear layer. INL: inner nuclear layer. GCL: ganglion cell layer. Scale bar: 20 µM. (B) Higher magnification images of (A). OS: outer segment. OONL: outer portion of the outer nuclear layer. IONL: inner portion of the outer nuclear layer. Scale bar: 15 µM. GFP positive (+) cells in ONL were counted in sections of retinas electroporated with shRNA targeting *Gapdh* or NRL target genes (C, F). Distribution of electroporated cell bodies in the retina (D). Fraction of GFP positive cells in the retinal outer nuclear layer is calculated. OONL, outer portion of outer nuclear layer; IONL, inner portion of outer nuclear layer. Average outer segment (OS) length of electroporated cells was measured (E). Data are represented as mean ± SD. (C, D, E) *P<0.001, **P<0.0001 by Student's t test (n = 6 electroporated retinas). (F) *P<0.01 by Student's t test (n = 3 electroporated retinas).

**Figure 7:**
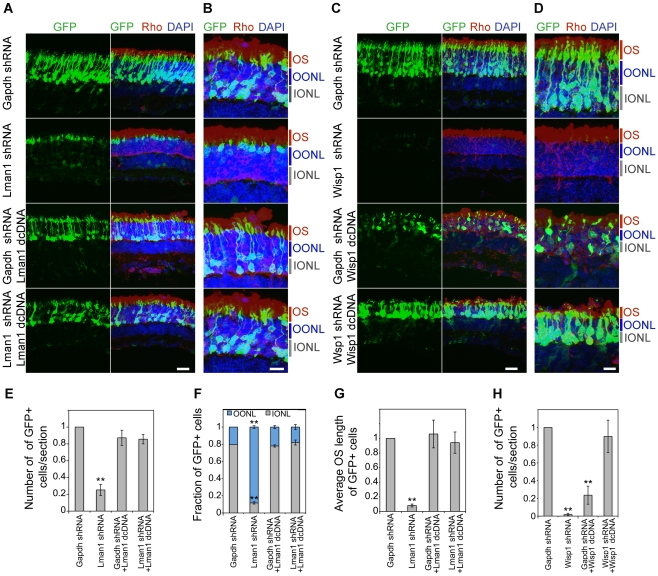
Rescue of shRNA–knockdown with shRNA–resistant dcDNA. CD-1 mice were transfected at P0 with Ub-GFP and shRNA against *Gapdh*, *Lman1 or Wisp1* by sub-retinal injection and *in vivo* electroporation. shRNA-resistant degenerate cDNA (dcDNA) was co-injected together with shRNA against *Gapdh*, *Lman1 or Wisp1* for rescue experiments. Retinas were harvested at P20 and examined for GFP fluorescence (green), Rho immuno-reactivity (red) and DAPI staining (blue). Three biological replicate retinas were collected and imaged. (A, C). Scale bar: 20 µM. (B, D) Higher magnification images of (A, C). OS: outer segment. OONL: outer portion of the outer nuclear layer. IONL: inner portion of the outer nuclear layer. Scale bar: 10 µM. GFP positive (+) cells were counted in sections of retina electroporated with shRNA targeting *Gapdh* or NRL target genes (E, H). Distribution of electroporated cell bodies in the retina (F). Fraction of GFP positive cells in the retinal outer nuclear layer is counted. OONL, outer region of outer nuclear layer; IONL, inner region of outer nuclear layer. Average outer segment (OS) lengths of electroporated cells were measured (G). Data are represented as mean ± SD. (E–H) *P<0.01, **P<0.001 by Student's t test (n = 3 electroporated retinas).

To directly examine the physiological function of 16 NRL target genes, we knocked down the expression of target genes by transfecting shRNA plasmids *in vivo* into the P0 mouse retina [44], [45]. For each target gene, three shRNA expression constructs were first evaluated for knockdown efficiency using a sensor construct in HEK293T cells (Figure S3). The most efficient shRNA was then used for *in vivo* knockdown experiments in the mouse retina, which were examined seven or twenty days (at P7 or P20) after electroporation (Figure 6, Figure 7, and Figures S4, S5). A GFP-expression plasmid (Ub-GFP) was co-transfected to mark the transfected retinal cells. Based on putative function and/or involvement in retinal disease (see Table S5), we selected 16 genes – *Bach2*, *Cdr2*, *Dusp12*, *Esrrb*, *Gpsm2*, *Haus1*, *Kdm5b*, *Lman1*, *Lrp11*, *Lrrc2*, *Ncoa2*, *Plekha2*, *Ppargc1b*, *Trim36*, *Wisp1 and Zdhhc14*. Eight of the genes have overlapping CRX ChIP–Seq peaks.

We consistently observed, in multiple biological replicates, smaller numbers of GFP+ cells in P20 retina that was transfected with shRNA against NRL target genes compared to the retina expressing control *Gapdh* shRNA (Figure 6, Figure 7, and Figure S5). The reduction in the number of GFP+ cells was more pronounced at P20 than at P7, and was most severe in retina transfected with *Wisp1* shRNA, which led to a near total and consistent loss of GFP+ cells at P20. Thus, the function of a majority of NRL targets appears to be required for functional maintenance of photoreceptors.

In addition to the reduced number of GFP+ cells, the knockdown of *Kdm5b*, *Lman1*, or *Wisp1* resulted in an abnormal morphology of the transfected photoreceptors at P20, including the abnormal location of their cell bodies (Figure 6A, 6B, and 6D) and short outer segments (Figure 6A, 6B, and 6E). The cell bodies of the GFP+ cells were positioned in the outer portion of the outer nuclear layer (ONL), reminiscent of cone nuclei [46], instead of spanning across the ONL (Figure 6).

To validate the specificity of knockdown data and rule out the possibility of general toxic effects of shRNA, we produced degenerate cDNA (dcDNA) constructs for two of the target genes (*Lman1* and *Wisp1*) containing silent mutations that conferred resistance to shRNA mediated mRNA degradation. Co-transfection of *Gapdh* shRNA with dcDNA for *Lman1* did not manifest a retinal phenotype, and more importantly, *Lman1* dcDNA co-transfection rescued all of the *Lman1* shRNA phenotypes in the retina (including the reduced number of GFP+ cells, cell body location and OS length) (Figure 7). Co-transfection with *Wisp1* dcDNA also corrected the reduction of GFP+ cells; however, its overexpression led to a decrease in GFP+ cells (Figure 7), indicating that endogenous WISP1 levels are carefully controlled.

### KDM5b Functions as a Secondary Regulatory Node

**Figure 8:**
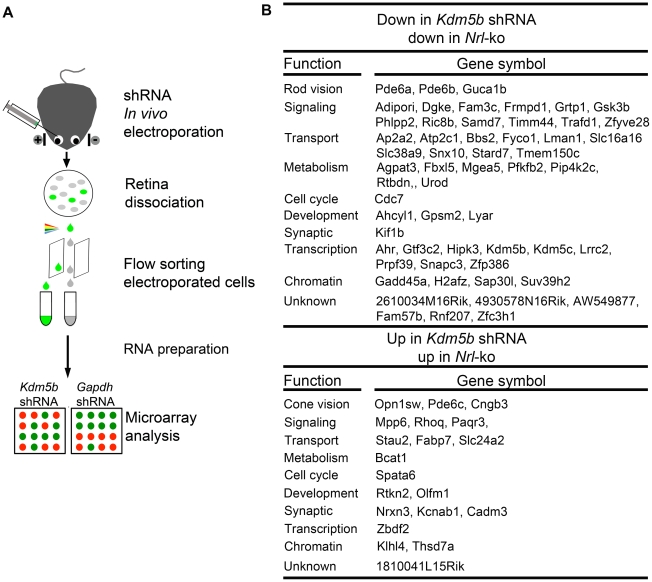
Microarray analysis of flow-sorted photoreceptors after *in vivo Kdm5b* knockdown. (A) Experimental workflow. CD-1 mouse retinas were transfected at P0 with Ub-GFP and shRNA against *Gapdh* or *Kdm5b* by sub-retinal injection and *in vivo* electroporation. Retinas were dissociated at P20 and shRNA-transfected retinal cells were isolated by flow-sorting. The effect of *Kdm5b* shRNA on transcriptional profile was measured by microarray analysis. (B) Ontology analysis of common targets of KDM5b and NRL. *Kdm5b* shRNA up: genes that are up-regulated in retinal cells electroporated with *Kdm5b* shRNA. *Kdm5b* shRNA down: genes that are down-regulated in retinal cells electroporated with *Kdm5b* shRNA. *Nrl-*ko up: genes that are up-regulated in *Nrl* ^−/−^ photoreceptor cells. *Nrl-*ko down: genes that are down-regulated in *Nrl* ^−/−^ photorceptor cells.

We were particularly intrigued by one of the NRL targets – *Kdm5b* (see Figure 6), which encodes lysine (K)-specific demethylase 5b, an enzyme that catalyzes the demethylation of active histone marks at methylated H3K4; thus, *Kdm5b* is involved in chromatin remodeling and functions as a transcriptional repressor [47], [48], [49]. To investigate its potential role as a second order node in photoreceptor GRN downstream of NRL, we dissociated the retina 20 days after knocking down *Kdm5b* or *Gapdh* expression by shRNA electroporation at P0, flow-sorted the electroporated cells, prepared total RNA, and performed global expression profiling using Affymetrix exon arrays (Figure 8A). *Kdm5b* knockdown resulted in up-regulation of 311 genes and down-regulation of 619 genes when compared to *Gapdh* knockdown. We detected 57 genes that are down-regulated and 20 that are up-regulated in both *Kdm5b* knockdown and *Nrl^−/−^* retina (Figure 8B), suggesting that some of the effects of loss of NRL (in *Nrl^−/−^* retina) are mediated through decreased *Kdm5b* expression. Some of the genes (e.g., *Pde6a*, *Pde6b*, *Guca1b*, *Pde6c*, *Cngb3*, *Opn1sw*) altered by *Kdm5b* knockdown are associated with the visual transduction, while a few others (*Gadd45a*, *H2afz* and *Suv39h2*) are associated with chromatin organization [50], [51], [52].

## Discussion

Visual impairment in a vast majority of retinal and macular degenerative diseases can be attributed to dysfunction or death of photoreceptors [7], [10], [11]. Despite the central role of cones in transduction of vision in humans, rods constitute 95% of all photoreceptors and are generally the first to die in retinal neurodegeneration. A relatively late onset of clinical manifestations in these diseases underscores the importance of stringently maintaining the function of highly metabolically active photoreceptors. The control of homeostasis must be exerted at multiple levels as quantitatively precise expression of phototransduction proteins and their transport to the modified sensory cilia (outer segments) are critical for photoreceptor survival. In addition to its essential role in photoreceptor differentiation, NRL has been implicated in the regulation of rod phototransduction genes, such as rhodopsin and cGMP phosphodiesterase α and β subunits [24], [27], [53], [54]. Here we identify global transcriptional targets of NRL and integrate our data with reported targets of CRX, another key regulator of photoreceptor genes. Our results show that NRL and CRX together control the expression of most, if not all, genes involved in rod phototransduction through a *cis*-regulatory module, which also includes the binding sites for NR2E3, ESRRβ, RORβ and in some cases MEF2C. Equally important is the finding that non-CRX containing NRL *cis*-regulatory modules fine-tune the expression of additional photoreceptor-expressed genes, which may contribute to high metabolic demand in rod photoreceptors.

ChIP–Seq has emerged as a cost effective, high-throughput technology for high-resolution genome-wide mapping of *in vivo* locations for chromatin modifications and transcription factor binding [55], [56], [57], [58]. Despite the fundamental difference in sequencing chemistry and nucleotide base calling software between the Illumina and ABI/SOLiD sequencing platforms [59], [60], our ChIP–Seq data from the two are remarkably comparable, further validating the *in vivo* NRL binding events reported here. In addition to enrichment in promoter regions, a number of NRL ChIP–Seq peaks are detected in intronic regions of annotated genes; some of these might reflect alternative promoter usage in photoreceptors as reported recently for *Mef2c* [39].

We previously proposed that photoreceptor precursors have a “default” S-cone fate and a “tug-of-war” among a selected few transcription factors specifies rod *versus* cone cell type [11]. NRL and TRβ2 respectively initiate the rod and M-cone pathways [16], with NRL being the dominant activator of rod genes and a suppressor of cone genes together with its target NR2E3 [15], [41]. Enrichment of a distinct set of transcription factor binding sites in NRL ChIP–Seq peaks in genes that are down- or up-regulated in *Nrl^−/−^* retina suggests specific and discrete *cis*-regulatory modules for rod *versus* cone photoreceptor expressed genes. CRX strongly activates the expression of both rod and cone genes [21], [22], [61]. An overlap of CRX peaks in over 50% of NRL ChIP–Seq peaks is consistent with their synergistic function in activating rod-expressed genes. Indeed, all rod phototransduction genes were included in this group. Notably, CRX ChIP–Seq peaks are much smaller than NRL peaks at the same loci and loss of NRL leads to more significant decrease in gene expression than in *Crx^−/−^* retina, suggesting a fundamental role of NRL in regulating rod genes. CRX likely enhances rod gene expression by altering the chromatin conformation *via* recruitment of histone acetylases [62]. In cone genes (up-regulated in *Nrl^−/−^* retina), binding of both CRX and NRL is consistent with the common photoreceptor precursor hypothesis [11], [16]. Additional studies (e.g., histone modifications) are needed to clarify differential regulation of specific genes by NRL and CRX in rod *versus* cone photoreceptors.

Like many key transcription factor nodes in GRNs [63], [64], [65], NRL likely auto-regulates its own expression as suggested by strong NRL ChIP–Seq peaks in *Nrl* promoter and intronic regions. While the key role of NR2E3 as a secondary node downstream of NRL is to repress cone-specific genes [17], [18], [41], two newly reported NRL targets – ESRRβ and MEF2C – function as transcriptional regulators for activation and/or maintenance of rod gene expression [38], [39]. A new secondary node in rod GRN that our studies identified is KDM5B (also called Jarid1b), a Jumonji-domain containing histone demethylase, which is associated with chromatin remodeling and transcriptional repression [47], [48]. KDM5B reportedly activates the expression of self-renewal-associated genes by suppressing cryptic initiation and maintaining proper H3K4me3 gradient for productive transcriptional elongation [66]. We observe a significant overlap between the genes altered by loss of NRL and KDM5B, indicating a broader role of KDM5B in regulating rod homeostasis downstream of NRL. We hypothesize that differential expression of KDM5B may contribute to chromatin organization and metabolic differences between rod and cone photoreceptors [8], [46], [67], [68].

Retinal and macular diseases are genetically heterogeneous with over 200 mapped loci; of these, almost 150 genes have been identified (http://www.sph.uth.tmc.edu/Retnet/). A catalog of genome-wide NRL targets with overlapping CRX binding sites, reported here, provides excellent candidate genes for mutation screening in patients with inherited retinal neurodegenerative diseases. We have listed almost 100 genes (see Table S5) that map to retinal disease loci. Interestingly, knockdown of 16 target genes, reported in this study, resulted in photoreceptor cell death or abnormal morphology, highlighting the importance of NRL targets in maintaining normal physiology and the association of perturbed target gene expression with retinal diseases.

A key aspect of photoreceptor homeostasis is the daily renewal of almost 10% of outer segment membrane discs, which requires a stringent control of the synthesis of specific phototransduction proteins and lipid molecules. Therefore, the target gene, *Lman1*, attracted our attention as its knockdown led to shorter photoreceptor outer segments and abnormal location of cell bodies (close to the sclera), which is characteristic of cone photoreceptors or late-born rods, whereas the early-born rods locate towards the vitreous side. LMAN1 participates in transport between the endoplasmic reticulum and Golgi [69]. Our data suggests that LMAN1 performs critical roles in photoreceptor homeostasis by controlling lipid homeostasis and/or biogenesis of membrane discs. Abnormal location of nuclei to scleral side in photoreceptors after its knockdown by *in vivo* electroporation could be due to rod to cone transformation in the absence of NRL, or delayed rod birth as a result of abnormal signaling for rod fate determination.

*Wisp1*, another interesting target of NRL, encodes the Wnt1-inducible signaling pathway protein 1 that exerts cytoprotective and/or growth promoting effects [70], [71] by repressing p53 and activation of Akt kinase [72]. WISP1 could therefore act as a survival or maintenance factor for photoreceptors. Further investigations on WISP1 may yield new targets for neuroprotective strategies in retinal degeneration.

**Figure 9:**
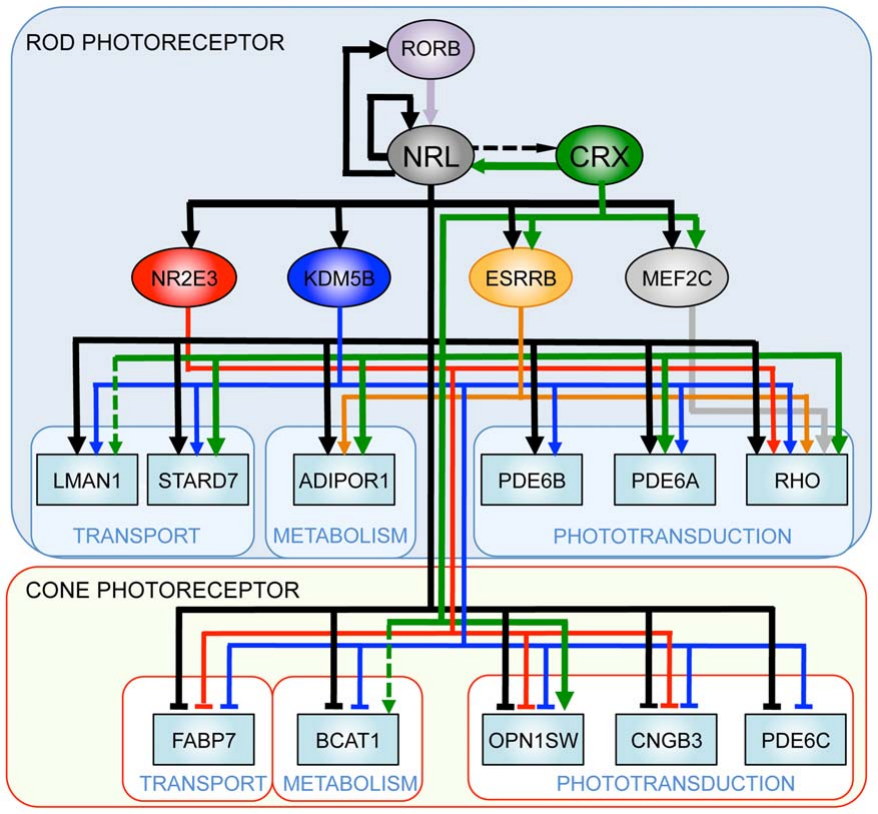
A simplified gene regulatory network in rod and cone photoreceptors. NRL acts synergistically with photoreceptor-specific transcription factors (such as CRX) and regulates the expression of rod and cone genes. Thin lines denote expression-based links, the dashed lines indicate links based on ChIP assays, and thick lines denote confirmed regulatory connections.

Gene regulatory networks (GRN) control multiple pathways during development and homeostasis and provide conceptual framework for elucidating disease mechanisms [2], [73]. Transcription factors reside near the top of GRNs; their abnormal expression and/or activity can cause widespread changes in target genes [74], [75]. Our studies demonstrate a pivotal role of NRL in controlling rod homeostasis by modulating the expression of numerous target genes, which in turn maintain distinct aspects of cell function and survival. Elucidation of combinatorial regulation of genes by NRL and its co-regulators (specifically CRX) and identification of distinct downstream nodes (such as KDM5B) provide a framework to construct GRN for functional maintenance in mammalian rod photoreceptors (Figure 9).

## Materials and Methods

All animal work must have been conducted according to relevant national and international guidelines. Animal Care and Use Committee of the National Eye Institute approved all mouse protocols. See also Text S1.

### ChIP–Seq by Illumina and ABI/SOLiD Platforms

Retina from postnatal day (P) 28 C57Bl/6J mice was used for ChIP experiments with NRL antibody or normal IgG, as previously described [41]. Fifteen or 25 ng of ChIP DNA from parallel experiments was used for library preparation and sequencing on Illumina 1G Genome Analyzer or ABI SOLiD V2 system, respectively.

### ChIP–Seq Data Analysis

Raw sequencing reads from Illumina or ABI platforms were mapped to the mouse genome (NCBI build 37) using Genomatix Mining Station (GMS).

ChIP–Seq peaks were called using MACS [28] and NGS-Analyzer (Genomatix). The union of overlapping peak regions from both methods was used in subsequent analyses.

### Comparison of NRL ChIP–Seq Peaks with CRX ChIP–Seq Data

The NRL ChIP–Seq peaks were compared to the CRX ChIP–Seq regions [22] using GenomeInspector (Genomatix; [31]).

### Gene Expression Analysis

Affymetrix microarray data from flow-sorted photoreceptors of WT and *Nrl* ^−/−^ retina [25] was analyzed using ChipInsepector (Genomatix).

### Transcription Factor Enrichment Analysis

Transcription factor binding site enrichment analyses of sequences in ChIP–Seq peak regions were performed using RegionMiner and MatInspector (Genomatix; [31], [32]). The ChIP–Seq peaks were extended to −500 bp and +500 bp from the peak center. The sequences were scanned for TFBS matrices (Genomatix MatBase version 8.2) using MatInspector (Genomatix). Positional bias (P) was calculated [76], and the –log(P) was plotted against the scan windows' mid-positions. The over-represented TFBS positions for a TF family appear as peaks in these plots.

### ChIP–Quantitative PCR (qPCR)

ChIP DNA was tested in triplicates by qPCR using SYBR Green [77]. We randomly tested 26 regions with peaks covering the majority range of the peak heights. Five regions without ChIP–Seq signals served as negative controls. Normal IgG served as the negative antibody control, and *Nrl* ^−/−^ retina was used as a negative tissue control. The complete ChIP-qPCR procedure was performed twice.

### Cell Culture, Transfection, Plasmids, and Cloning

HEK293T cells were cultured in DMEM and transfected with Fugene 6 (Roche).

To generate shRNA-resistant dcDNA constructs, silent mutations that confer resistance to shRNA were introduced into *Lman1* cDNA and *Wisp1* cDNA using Quikchange kit (Stratagene).

### Enhancer Analysis Using Luciferase Assays

To generate enhancer constructs, ChIP–Seq peak regions were amplified and cloned into pGL3-promoter vector (Promega). HEK293T cells were transfected with these enhancer constructs, a transfection control plasmid expressing Renilla luciferase (Promega), and NRL expression plasmid or empty vector. The luciferase activities were measured 48 hr after transfection. The experiments were performed three times.

### shRNA–Sensor Assay

To generate shRNA-sensor constructs for efficiency test, we cloned the shRNA target sequences into the 3′UTR of a GFP vector. The shRNA-sensor construct and CAG-HcRed (transfection control) were co-transfected with either shRNA against target or *Gapdh* shRNA, For each target gene, three shRNA constructs were evaluated for efficacy indicated by a decrease in GFP. The most efficient one was chosen for *in vivo* knockdown experiments.

### Sub-Retinal Injection and *In Vivo* Electroporation

shRNA alone or together with shRNA-resistant dcDNA was introduced in the retina of CD-1 P0 mouse pups by sub-retinal injection followed by *in vivo* electroporation, as previously described [44], [45]. The retinas were harvested at P7 or P20 for histology or immunohistochemistry.

### Retina Dissociation, FACS Isolation, and Exon Arrays

Mouse retina was electroporated at P0 with Ub-GFP and *Gapdh* shRNA or *Kdm5b* shRNA and dissected at P20. GFP+ retinal cells were isolated from dissociated retina by FACS (FACSAria; BD Biosciences). RNA was extracted and cDNA was synthesized followed by sense transcript cDNA (ST-cDNA) generation using WT-Ovation Exon module (NuGEN Technologies). The ST-cDNA was fragmented and labeled with Encore Biotin Module (NuGen) and used for hybridization with GeneChip Mouse Exon 1.0 ST array (Affymetrix). The microarray data has been deposited in the Gene Expression Omnibus Database (accession #: will be available soon).

## Supporting Information

Figure S1 Genomic distribution of NRL ChIP–Seq peaks that are enriched for distinct TF families. NRL ChIP–Seq peak regions (Illumina), enriched for the top 8 TF families (Table S1), were mapped to the nearest annotated genes. Down: ChIP–Seq peaks associated with genes down-regulated in *Nrl*-ko mouse photoreceptors (mRNA level decreased ≥1.5 fold in Affymetrix analysis). Up: ChIP–Seq peaks associated with genes up-regulated in *Nrl*-ko mouse photoreceptors (mRNA level increased ≥1.5 fold in Affymetrix analysis).
(TIF)

Figure S2 Enhancer function of NRL ChIP–Seq regions associated with cone genes in transfected HEK293T cells. NRL peak regions associated with cone genes (*Gnat2*, m-Opsin, *Gngt2* and *Pik3ap1*) were cloned into pGL3-promoter vector in front of SV40 basal promoter and a luciferase reporter. The enhancer constructs (200 ng) were transfected in HEK293T cells with increasing amount of mouse *Nrl* (m*Nrl*) expression plasmid (in pC4C vector). Empty pC4C vector was included to make the total amount of DNA equal among different transfection groups. The y-axis shows fold change (FC) of normalized luciferase readings. Two non-peak regions served as additional negative controls. The experiments were performed three times, and the representative results are shown as mean ± SD. * P<0.01 by Student's t test.
(TIF)

Figure S3 Efficacy tests for shRNA constructs, using a reporter assay in HEK293T cells. Sensor constructs included shRNA target sequences in 3′UTR of GFP. The *Gapdh* shRNA or target gene shRNA construct was co-transfected with the sensor construct and CAG-HcRed into HEK293T cells. Sensor knockdown was imaged at 48 h after transfection.
(TIF)

Figure S4 Effects of *in vivo* knockdown of *Kdm5b*, *Lman1* or *Wisp1* in P0 retina evaluated at P7. Ub-GFP and shRNA against *Gapdh*, *Kdm5b*, *Lman1* or *Wisp1* were co-injected in the sub-retinal space of CD-1 mice at postnatal day 0 (P0), followed by electroporation. Retinas were harvested at P7 and examined for GFP (green) fluorescence and Rho (red) and DAPI (blue) staining. Scale bar: 20 µm.
(TIF)

Figure S5 *In vivo* knockdown of additional NRL target genes. Ub-GFP and shRNA against 13 NRL target genes (*Bach2*, *Cdr2*, *Dusp12*, *Esrrb*, *Gpsm2*, *Haus1*, *Lrp11*, *Lrrc2*, *Ncoa2*, *Ppargc1b*, *Trim36*, *Plekha2*, *Zdhhc14*) *or Gapdh* were injected in the sub-retinal space of CD-1 mice at P0 and electroporation was performed. Retinas were harvested at P20 and examined for GFP (green) fluorescence and Rho (red) and DAPI (blue) staining. Scale bar: 20 µm.
(TIF)

Table S1 Genomic distribution of NRL ChIP–Seq peaks relative to the nearest TSS. Genomic distribution of NRL ChIP–Seq peaks was categorized according to the distance to the nearest TSS. TSS: transcription start site. %: percentage.
(DOCX)

Table S2 TF enrichment analysis of NRL ChIP–Seq peaks associated with altered genes (*Nrl*-ko vs. wt photoreceptors). Genomatix software was used to perform an unbiased analysis of sequences +500 bp from the NRL ChIP–Seq peak center for over-represented TFs. The commonly enriched TFs in Illumina and ABI data are shown. The number of input sequences with match, matches in input from ABI data were shown. The enriched TFs are ranked by Z scores, and TFs in gray are not significantly enriched (Z score<2). Down in *Nrl*-ko: ChIP–Seq peaks associated with genes down-regulated in *Nrl*-ko mouse photoreceptors (mRNA level decreased>1.5 fold in Affymetrix analysis). Up in *Nrl*-ko: ChIP–Seq peaks associated with genes up-regulated in *Nrl*-ko mouse photoreceptors (mRNA level increased>1.5 fold in Affymetrix analysis). *Nrl*-ko: *Nrl* knock out mice. wt: wild type mice.
(XLSX)

Table S3 TF enrichment analysis of CRX-overlapping or non CRX-overlapping NRL ChIP–Seq peaks associated with genes down-regulated in *Nrl*-ko photoreceptors. An unbiased analysis of sequences that are +500 bp from the NRL ChIP–Seq peak center was performed for over-represented TFs using Genomatix software. The TFs identified in both Illumina and ABI data are shown. The enriched TFs are ranked by Z scores. TFs in gray are not significantly enriched (Z score<2). Down in *Nrl*-ko: ChIP–Seq peaks associated with down-regulated genes in *Nrl*-ko mouse photoreceptors (mRNA level decreased>1.5 fold in Affymetrix analysis). Overlap with CRX: NRL ChIP–Seq peaks that overlap with CRX ChIP–Seq peaks. Non overlap with CRX: NRL ChIP–Seq peaks that do not overlap with CRX ChIP–Seq peaks. *Nrl*-ko: Nrl knock out mice.
(DOC)

Table S4 Top 40 biological processes associated with genes at or near the CRX-overlapping or non CRX-overlapping NRL ChIP–Seq peaks. Genomatix software was used to perform an unbiased analysis of biological processes that are associated with the genes at/near the CRX-overlapping or non CRX-overlapping NRL ChIP–Seq peaks. The top 40 biological processes in Illumina and ABI data are shown. Overlap with CRX: NRL ChIP–Seq peaks that overlap with CRX ChIP–Seq peaks. Non-overlap with CRX: NRL ChIP–Seq peaks that do not overlap with CRX ChIP–Seq peaks. Photoreceptor-related/specific biological processes are highlighted in green.
(DOC)

Table S5 Identification of candidate retinal disease genes. Candidate retinal disease genes are based on the chromosomal location of the human orthologs of the NRL target genes that map within a mapped retinal disease locus reported in the RetNet database (www.sph.uth.tmc.edu/retnet/). The top part of the table lists the retinal disease genes that have been identified, whereas the bottom part lists the candidate genes in the region of uncloned but mapped disease locus. The fold enrichment value was produced by dividing the total number of genes (reported in RefSeq) within each locus by the number of NRL target genes at the same locus.
(DOC)

Text S1 Supplemental extended experimental procedures.
(DOC)
