# Supplementary figures and images for: Transcriptional Regulation of Rod Photoreceptor Homeostasis Revealed by In Vivo NRL Targetome Analysis
Source: PLoS Genet. 2012 Apr 12;8(4):e1002649. doi: 10.1371/journal.pgen.1002649 (PMC3325202; doi:10.1371/journal.pgen.1002649)

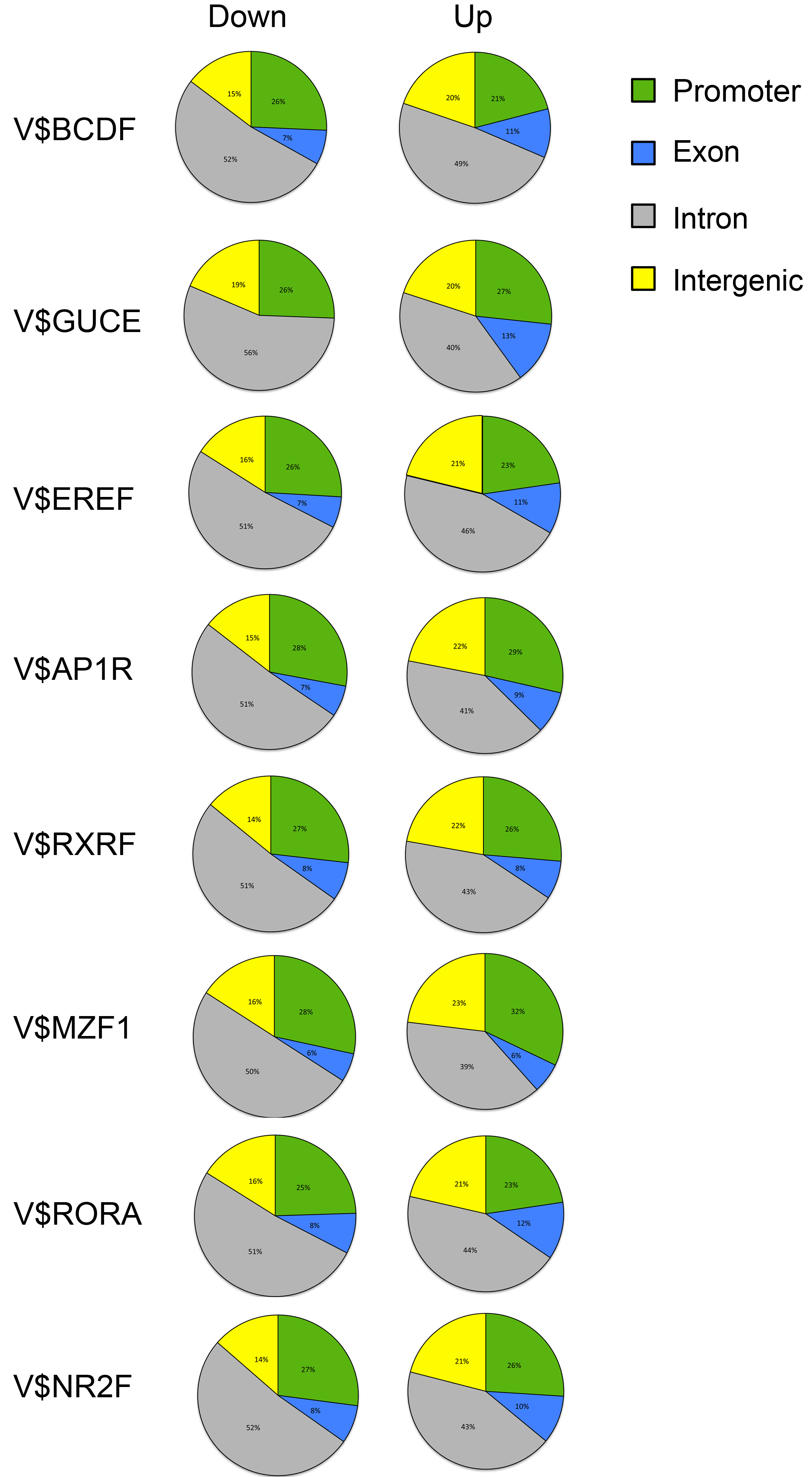

Supplement: Figure S1 — Genomic distribution of NRL ChIP–Seq peaks that are enriched for distinct TF families. NRL ChIP–Seq peak regions (Illumina), enriched for the top 8 TF families (Table S1), were mapped to the nearest annotated genes. Down: ChIP–Seq peaks associated with genes down-regulated in Nrl-ko mouse photoreceptors (mRNA level decreased ≥1.5 fold in Affymetrix analysis). Up: ChIP–Seq peaks associated with genes up-regulated in Nrl-ko mouse photoreceptors (mRNA level increased ≥1.5 fold in Affymetrix analysis). (TIF) [file pgen.1002649.s001.tif]

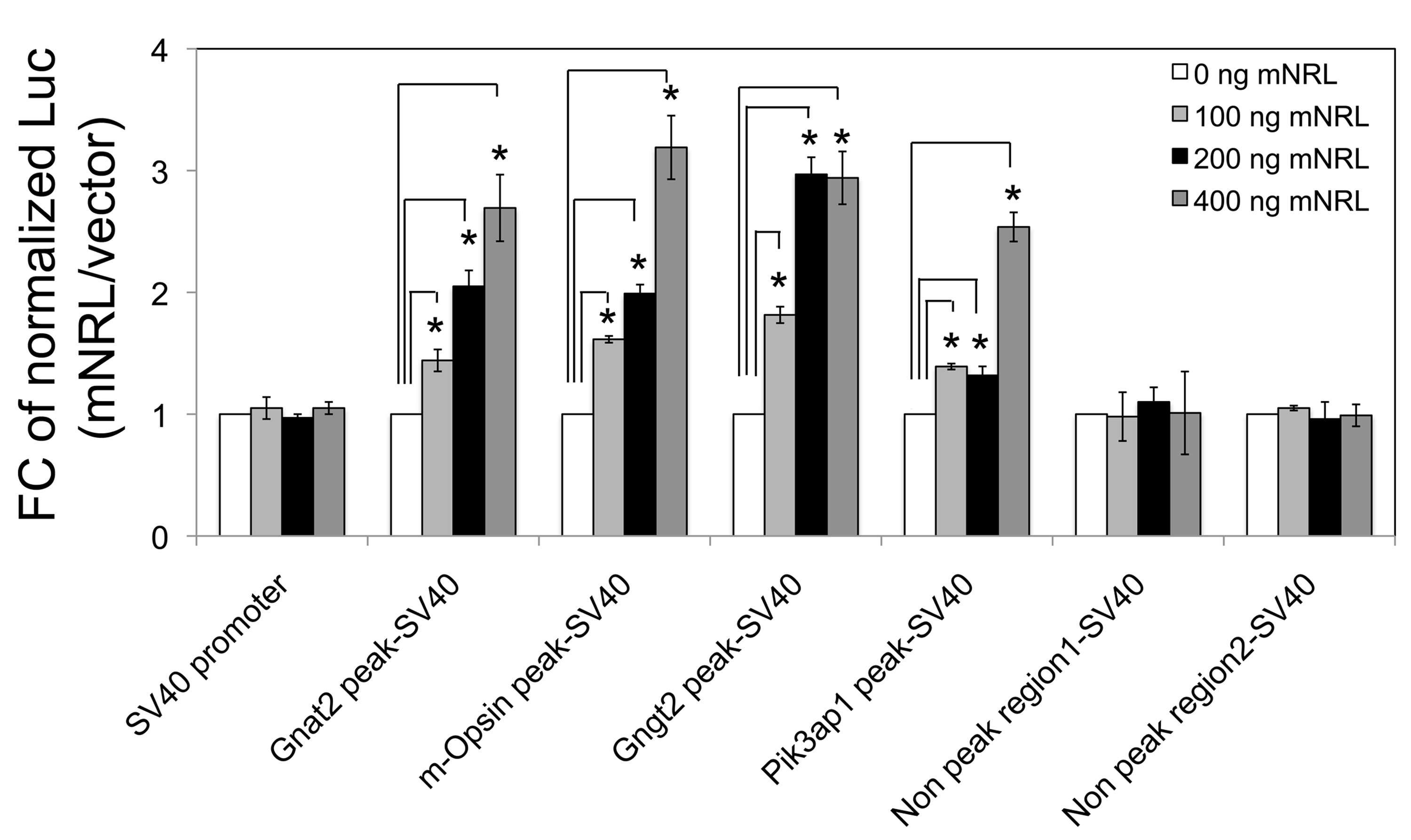

Supplement: Figure S2 — Enhancer function of NRL ChIP–Seq regions associated with cone genes in transfected HEK293T cells. NRL peak regions associated with cone genes (Gnat2, m-Opsin, Gngt2 and Pik3ap1) were cloned into pGL3-promoter vector in front of SV40 basal promoter and a luciferase reporter. The enhancer constructs (200 ng) were transfected in HEK293T cells with increasing amount of mouse Nrl (mNrl) expression plasmid (in pC4C vector). Empty pC4C vector was included to make the total amount of DNA equal among different transfection groups. The y-axis shows fold change (FC) of normalized luciferase readings. Two non-peak regions served as additional negative controls. The experiments were performed three times, and the representative results are shown as mean ± SD. * P<0.01 by Student's t test. (TIF) [file pgen.1002649.s002.tif]

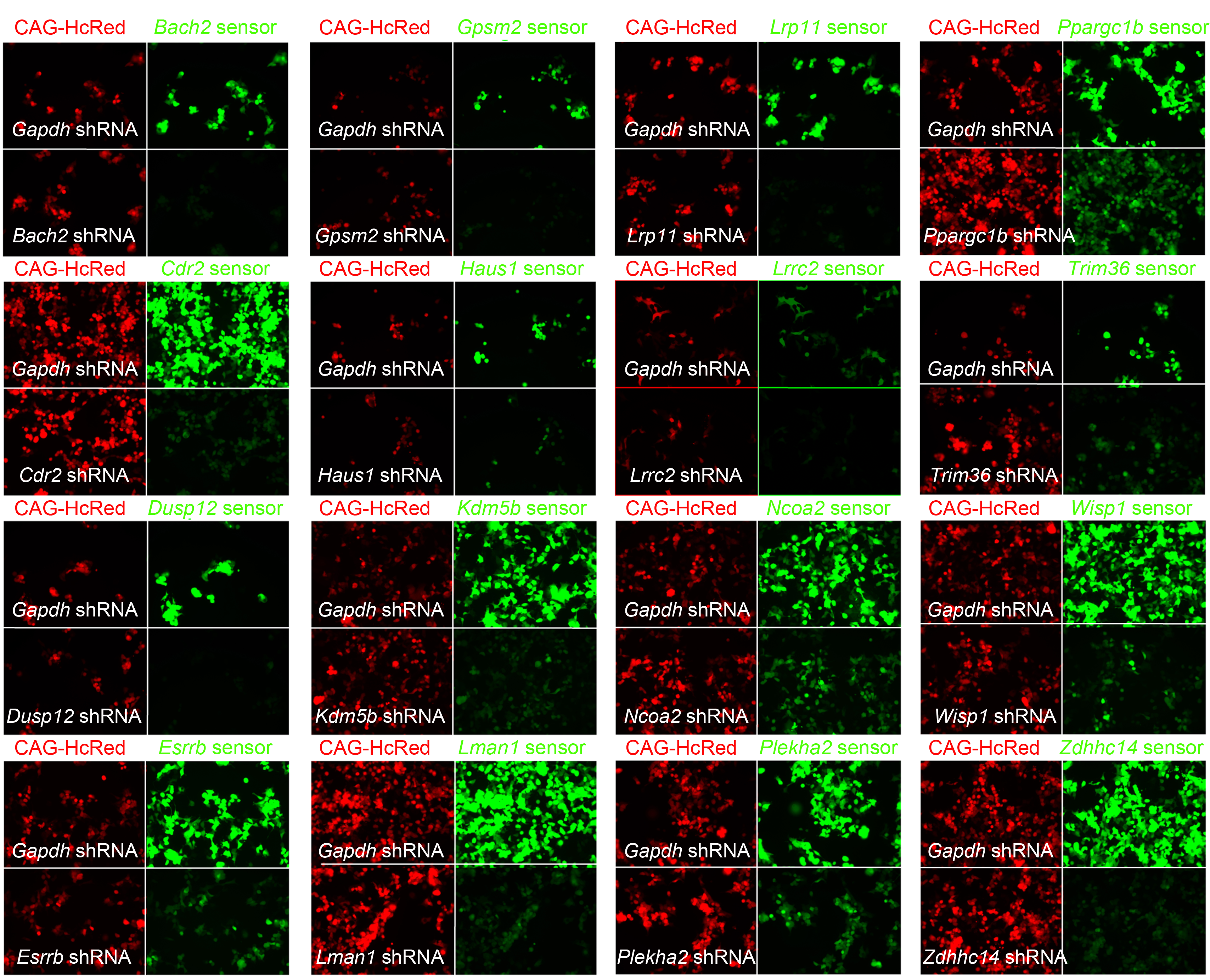

Supplement: Figure S3 — Efficacy tests for shRNA constructs, using a reporter assay in HEK293T cells. Sensor constructs included shRNA target sequences in 3′UTR of GFP. The Gapdh shRNA or target gene shRNA construct was co-transfected with the sensor construct and CAG-HcRed into HEK293T cells. Sensor knockdown was imaged at 48 h after transfection. (TIF) [file pgen.1002649.s003.tif]

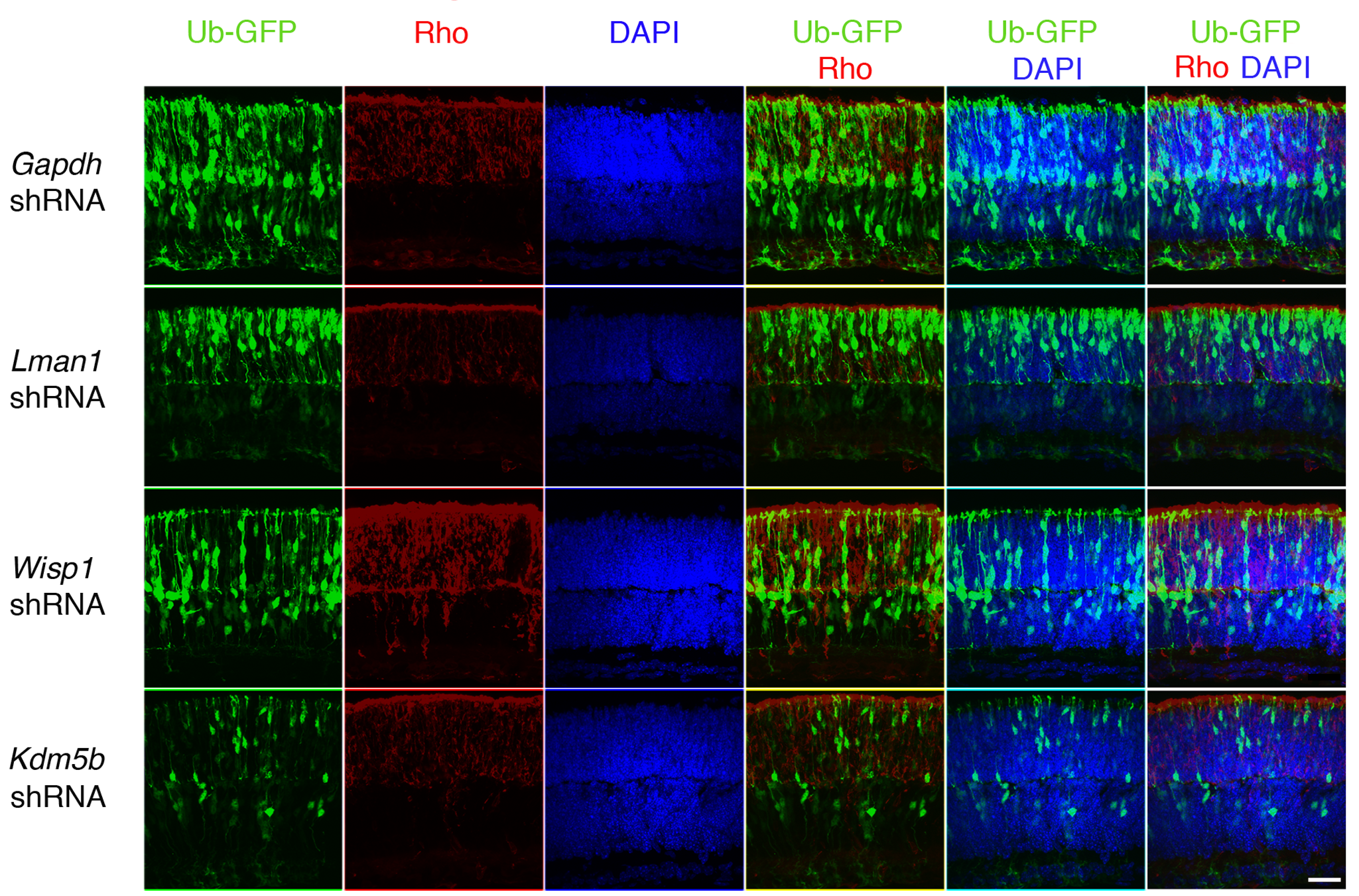

Supplement: Figure S4 — Effects of in vivo knockdown of Kdm5b, Lman1 or Wisp1 in P0 retina evaluated at P7. Ub-GFP and shRNA against Gapdh, Kdm5b, Lman1 or Wisp1 were co-injected in the sub-retinal space of CD-1 mice at postnatal day 0 (P0), followed by electroporation. Retinas were harvested at P7 and examined for GFP (green) fluorescence and Rho (red) and DAPI (blue) staining. Scale bar: 20 µm. (TIF) [file pgen.1002649.s004.tif]

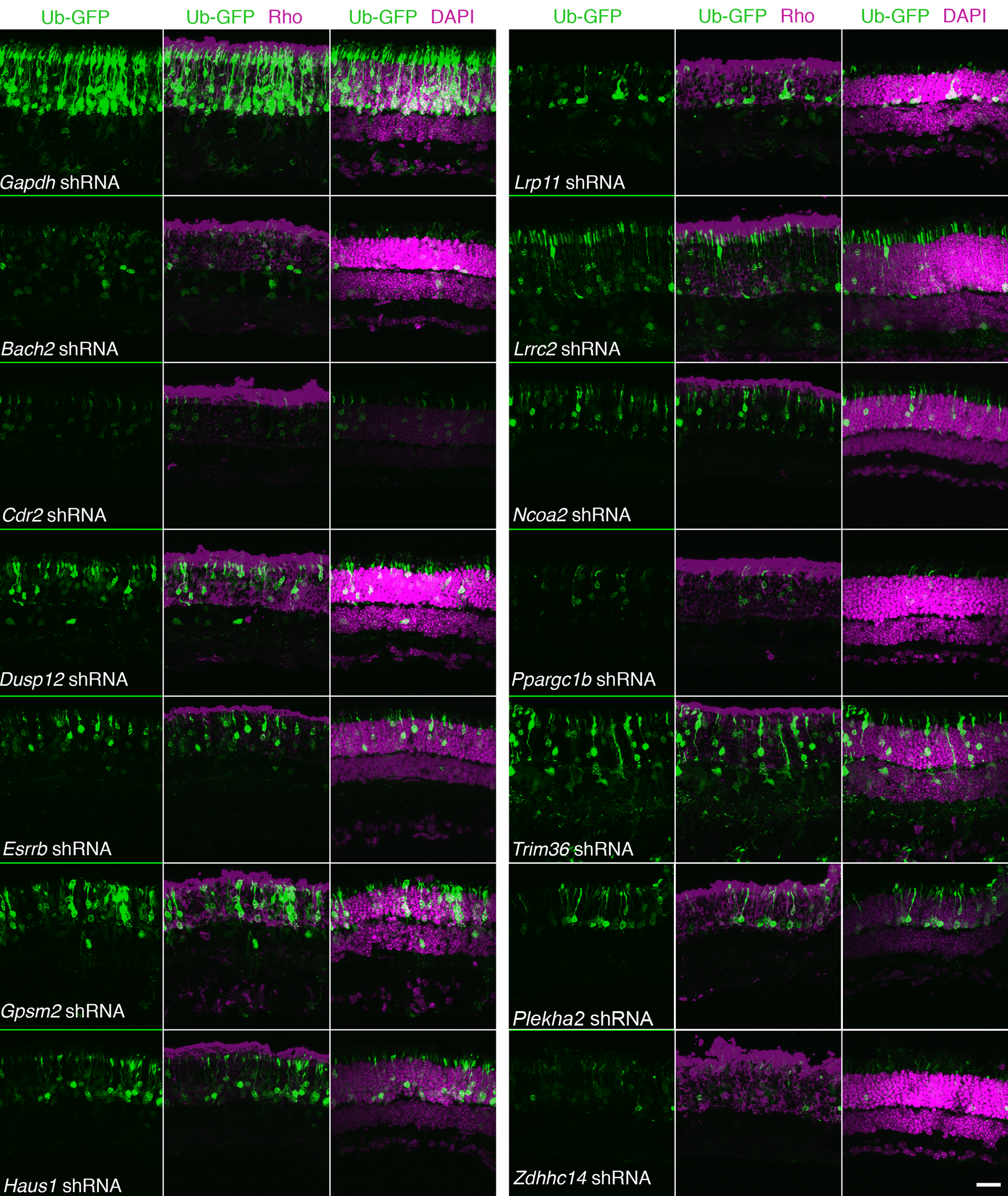

Supplement: Figure S5 — In vivo knockdown of additional NRL target genes. Ub-GFP and shRNA against 13 NRL target genes (Bach2, Cdr2, Dusp12, Esrrb, Gpsm2, Haus1, Lrp11, Lrrc2, Ncoa2, Ppargc1b, Trim36, Plekha2, Zdhhc14) or Gapdh were injected in the sub-retinal space of CD-1 mice at P0 and electroporation was performed. Retinas were harvested at P20 and examined for GFP (green) fluorescence and Rho (red) and DAPI (blue) staining. Scale bar: 20 µm. (TIF) [file pgen.1002649.s005.tif]
