# Supplementary material for: Transcriptional Regulation of Rod Photoreceptor Homeostasis Revealed by In Vivo NRL Targetome Analysis
Source: PLoS Genet. 2012 Apr 12;8(4):e1002649. doi: 10.1371/journal.pgen.1002649 (PMC3325202; doi:10.1371/journal.pgen.1002649)
Supplement: Table S1 — Genomic distribution of NRL ChIP–Seq peaks relative to the nearest TSS. Genomic distribution of NRL ChIP–Seq peaks was categorized according to the distance to the nearest TSS. TSS: transcription start site. %: percentage. (DOCX) [file pgen.1002649.s006.docx]

**Table S1. Genomic distribution of NRL ChIP-Seq peaks relative to the nearest TSS**

|  | **ABI** | | **Illumina** | |
| --- | --- | --- | --- | --- |
| **Distance to TSS** | **%** | **Median P value** | **%** | **Median P value** |
| Over 3 kb upstream | 31.4 | < 1 x 10^-6^ | 35.1 | < 1.6 x 10^-6^ |
| 2 kb to 3 kb upstream | 3.4 | < 1 x 10^-7^ | 3.9 | < 1.8 x 10^-6^ |
| 1 kb to 2 kb upstream | 4.2 | < 1.8 x 10^-6^ | 4.7 | < 3.4 x 10^-6^ |
| Within 1 kb upstream | 17.6 | < 1 x 10^-6^ | 14.3 | < 1 x 10^-5^ |
| Within 1 kb downstream | 19.6 | < 1 x 10^-6^ | 14.7 | < 2.9 x 10^-6^ |
| 1 kb to 2 kb downstream | 2.7 | < 1.7 x 10^-7^ | 3 | < 1 x 10^-5^ |
| 2 kb to 3 kb downstream | 1.8 | < 3.5 x10^-8^ | 2.5 | < 1.6 x 10^-6^ |
| Over 3 kb downstream | 19.3 | < 3.6 x 10^-7^ | 21.9 | < 1.8 x 10^-6^ |

Genomic distribution of NRL ChIP-Seq peaks was categorized according to the distance to the nearest TSS. TSS: transcription start site. %: percentage.
