# Supplementary material for: Transcriptional Regulation of Rod Photoreceptor Homeostasis Revealed by In Vivo NRL Targetome Analysis
Source: PLoS Genet. 2012 Apr 12;8(4):e1002649. doi: 10.1371/journal.pgen.1002649 (PMC3325202; doi:10.1371/journal.pgen.1002649)
Supplement: Table S3 — TF enrichment analysis of CRX-overlapping or non CRX-overlapping NRL ChIP–Seq peaks associated with genes down-regulated in Nrl-ko photoreceptors. An unbiased analysis of sequences that are +500 bp from the NRL ChIP–Seq peak center was performed for over-represented TFs using Genomatix software. The TFs identified in both Illumina and ABI data are shown. The enriched TFs are ranked by Z scores. TFs in gray are not significantly enriched (Z score<2). Down in Nrl-ko: ChIP–Seq peaks associated with down-regulated genes in Nrl-ko mouse photoreceptors (mRNA level decreased>1.5 fold in Affymetrix analysis). Overlap with CRX: NRL ChIP–Seq peaks that overlap with CRX ChIP–Seq peaks. Non overlap with CRX: NRL ChIP–Seq peaks that do not overlap with CRX ChIP–Seq peaks. Nrl-ko: Nrl knock out mice. (DOC) [file pgen.1002649.s008.doc]

**Table S3.** TF enrichment analysis of CRX-overlapping or non CRX-overlapping NRL ChIP-Seq peaks associated with genes down-regulated in *Nrl*-ko photoreceptors

|  |  |  | **Overlap with CRX** | | **Non overlap with CRX** | |
| --- | --- | --- | --- | --- | --- | --- |
| **TF family** | **TF family full name** | **Representative TF** | **Z score** | **Rank** | **Z score** | **Rank** |
| V$BCDF | Bicoid-like homeodomain transcription factors | Crx, Otx2 | 13.4 | 1 | #N/A | #N/A |
| V$AP1R | MAF and AP1 related factors | Bach2, Nrl | 12.3 | 2 | 4.6 | 8 |
| V$GUCE | GTF2IRDI upstream control element | Gtf2ird1, Gtf2i | 11.8 | 3 | #N/A | #N/A |
| V$EREF | Estrogen response elements | Esrra, Esrrb | 6.1 | 4 | -0.7 | 94 |
| V$RORA | v-ERB and RAR-related orphan receptor alpha | Rora, Rorb | 5.3 | 5 | -1.6 | 124 |
| V$RXRF | RXR heterodimer binding sites | Rxra, Rxrb | 4.6 | 6 | 0.4 | 54 |
| V$NR2F | Nuclear receptor subfamily 2 factors | Nr2e3, Nr2f1 | 3.5 | 7 | -1.9 | 128 |
| V$SF1F | Vertebrate steroidogenic factor | Nr5a1, Nr5a2 | 3.3 | 9 | -0.8 | 98 |
| V$MEF3 | MEF3 binding sites | Six2, Six5 | 2.9 | 10 | -1.6 | 123 |
| V$CSEN | Calsenilin, presenilin binding protein, EF hand factors | Kcnip3, Kcnip2 | 2.2 | 11 | -0.9 | 100 |
| V$PERO | Peroxisome proliferator-activated receptor | Rxra, Pparg | 2.2 | 12 | -0.6 | 93 |
| V$FXRE | Farnesoid X - activated receptor response elements | Rxra, Rxrb | 2.2 | 13 | 0.7 | 48 |
| V$ZNFP | Zinc finger proteins | Szf1 | 2.0 | 15 | 0.8 | 43 |
| V$MYOD | Myoblast determining factors | Ascl3, Tcf4 | 1.9 | 18 | 3.4 | 14 |
| V$YBXF | Y-box binding transcription factors, multifunction factors | Ybx1, Csda | 1.4 | 28 | 3.5 | 13 |
| V$MZF1 | Myeloid zinc finger 1 factors | Mzf1 | 1.2 | 33 | 4.5 | 9 |
| V$AP2F | Activator protein 2 | Tcfap2a, Tcfap2b | 0.7 | 49 | 3.9 | 11 |
| V$PAX5 | PAX-5 B-cell-specific activator protein | Pax5 | -0.9 | 96 | 4.6 | 7 |
| V$AHRR | AHR-arnt heterodimers and AHR-related factors | Ahr, Npas4 | -2.2 | 126 | 2.5 | 17 |
| V$PLAG | Pleomorphic adenoma gene | Plag1, Plagl1 | -2.5 | 135 | 2.8 | 16 |
| V$SP1F | GC-Box factors SP1/GC | Sp1, Sp3 | -3.1 | 143 | 6.8 | 2 |
| V$KLFS | Krueppel like transcription factors | Klf4, Klf9 | -3.1 | 144 | 6.0 | 3 |
| V$CTCF | CTCF and BORIS gene family | Ctcf, Ctcfl | -3.7 | 156 | 5.1 | 4 |
| V$MAZF | Myc associated zinc fingers | Maz, Ptz1 | -3.9 | 163 | 4.4 | 10 |
| V$EGRF | EGR/nerve growth factor induced proteins | Egr1, Egr2 | -6.0 | 177 | 4.8 | 6 |

An unbiased analysis of sequences that are +500 bp from the NRL ChIP-Seq peak center was performed for over-represented TFs using Genomatix software. The TFs identified in both Illumina and ABI data are shown. The enriched TFs are ranked by Z scores. TFs in gray are not significantly enriched (Z score < 2). **Down in *Nrl*-ko:** ChIP-Seq peaks associated with down-regulated genes in *Nrl*-ko mouse photoreceptors (mRNA level decreased > 1.5 fold in Affymetrix analysis). Overlap with CRX: NRL ChIP-Seq peaks that overlap with CRX ChIP-Seq peaks. Non overlap with CRX: NRL ChIP-Seq peaks that do not overlap with CRX ChIP-Seq peaks. *Nrl*-ko: Nrl knock out mice.
