# Supplementary material for: Transcriptional Regulation of Rod Photoreceptor Homeostasis Revealed by In Vivo NRL Targetome Analysis
Source: PLoS Genet. 2012 Apr 12;8(4):e1002649. doi: 10.1371/journal.pgen.1002649 (PMC3325202; doi:10.1371/journal.pgen.1002649)
Supplement: Table S4 — Top 40 biological processes associated with genes at or near the CRX-overlapping or non CRX-overlapping NRL ChIP–Seq peaks. Genomatix software was used to perform an unbiased analysis of biological processes that are associated with the genes at/near the CRX-overlapping or non CRX-overlapping NRL ChIP–Seq peaks. The top 40 biological processes in Illumina and ABI data are shown. Overlap with CRX: NRL ChIP–Seq peaks that overlap with CRX ChIP–Seq peaks. Non-overlap with CRX: NRL ChIP–Seq peaks that do not overlap with CRX ChIP–Seq peaks. Photoreceptor-related/specific biological processes are highlighted in green. (DOC) [file pgen.1002649.s009.doc]

**Table S4.** Top 40 biological processes associated with genes at or near the CRX-overlapping or non CRX-overlapping NRL ChIP-Seq peaks

| **Illumina** |  |  |  |
| --- | --- | --- | --- |
| **Overlap w CRX** |  | **Non-overlap w CRX** |  |
| **GO-Term** | **P-value** | **GO-Term** | **P-value** |
| visual perception | 2.90E-31 | cellular process | 4.03E-10 |
| sensory perception of light stimulus | 4.46E-31 | cellular component organization | 1.61E-09 |
| cellular process | 3.52E-16 | regulation of metabolic process | 1.13E-08 |
| cellular metabolic process | 3.55E-14 | regulation of cellular metabolic process | 1.20E-08 |
| metabolic process | 5.93E-14 | regulation of primary metabolic process | 2.10E-08 |
| detection of light stimulus | 3.85E-13 | cellular metabolic process | 4.98E-08 |
| phototransduction | 6.01E-13 | biological regulation | 5.58E-08 |
| primary metabolic process | 6.53E-13 | primary metabolic process | 6.76E-08 |
| cellular component organization | 1.21E-12 | cellular biosynthetic process | 7.24E-08 |
| response to light stimulus | 1.56E-12 | cellular macromolecule biosynthetic process | 9.81E-08 |
| photoreceptor cell development | 3.57E-12 | metabolic process | 1.36E-07 |
| localization | 1.50E-11 | regulation of macromolecule metabolic process | 1.39E-07 |
| transport | 2.86E-11 | macromolecule biosynthetic process | 1.69E-07 |
| detection of abiotic stimulus | 3.78E-11 | biosynthetic process | 1.85E-07 |
| establishment of localization | 4.71E-11 | regulation of biological process | 2.85E-07 |
| photoreceptor cell differentiation | 6.21E-11 | cellular macromolecule metabolic process | 2.88E-07 |
| neuron development | 6.69E-11 | regulation of macromolecule biosynthetic process | 4.08E-07 |
| eye photoreceptor cell development | 1.78E-10 | gene expression | 5.30E-07 |
| response to radiation | 2.16E-10 | regulation of cellular biosynthetic process | 5.49E-07 |
| detection of external stimulus | 2.35E-10 | regulation of biosynthetic process | 7.01E-07 |
| generation of neurons | 5.81E-10 | nucleobase, nucleotide and nucleic acid metabolism | 7.56E-07 |
| response to stimulus | 6.19E-10 | regulation of gene expression | 7.64E-07 |
| neuron differentiation | 6.28E-10 | nitrogen compound metabolic process | 1.00E-06 |
| eye photoreceptor cell differentiation | 8.31E-10 | regulation of nitrogen compound metabolism | 1.03E-06 |
| response to abiotic stimulus | 1.32E-09 | regulation of cellular process | 1.46E-06 |
| detection of stimulus | 1.48E-09 | nucleic acid metabolic process | 2.31E-06 |
| sensory organ development | 1.71E-09 | macromolecule metabolic process | 2.31E-06 |
| eye morphogenesis | 2.03E-09 | cellular nitrogen compound metabolism | 2.56E-06 |
| cell development | 3.32E-09 | nucleobase, nucleotide and nucleic acid metabolism | 3.91E-06 |
| eye development | 4.51E-09 | transcription | 4.62E-06 |
| neurogenesis | 4.56E-09 | organelle organization | 4.69E-06 |
| cell projection organization | 1.06E-08 | regulation of transcription | 6.88E-06 |
| detection of visible light | 1.13E-08 | positive regulation of cellular metabolic process | 7.43E-06 |
| nervous system development | 2.54E-08 | positive regulation of nucleic acid metabolism | 8.79E-06 |
| macromolecule localization | 4.91E-08 | positive regulation of nitrogen compound mmetabolism | 1.36E-05 |
| nucleobase, nucleotide and nucleic acid metabolism | 7.89E-08 | positive regulation of macromolecule metabolism | 1.43E-05 |
| cellular macromolecule metabolic process | 8.14E-08 | positive regulation of metabolic process | 1.88E-05 |
| regulation of cellular metabolic process | 1.02E-07 | positive regulation of cellular process | 2.30E-05 |
| regulation of nitrogen compound metabolism | 1.08E-07 | cellular component assembly | 3.71E-05 |
| cellular localization | 1.51E-07 | positive regulation of transcription | 4.56E-05 |
| **ABI** |  |  |  |
| **Overlap w CRX** |  | **Non-overlap w CRX** |  |
| **GO-Term** | **P-value** | **GO-Term** | **P-value** |
| cellular metabolic process | 1.02E-24 | cellular process | 7.02E-25 |
| metabolic process | 1.03E-23 | cellular metabolic process | 1.50E-24 |
| cellular process | 7.96E-23 | metabolic process | 1.30E-22 |
| visual perception | 7.40E-22 | primary metabolic process | 3.31E-21 |
| sensory perception of light stimulus | 1.08E-21 | cellular macromolecule metabolic process | 3.68E-17 |
| primary metabolic process | 3.26E-21 | macromolecule metabolic process | 6.06E-16 |
| localization | 3.95E-21 | cellular biosynthetic process | 2.22E-15 |
| transport | 4.78E-21 | biosynthetic process | 2.83E-15 |
| establishment of localization | 5.51E-21 | regulation of metabolic process | 1.48E-14 |
| cellular component organization | 1.37E-18 | cellular protein metabolic process | 2.30E-13 |
| biosynthetic process | 3.63E-17 | regulation of cellular metabolic process | 3.32E-13 |
| cellular biosynthetic process | 8.55E-17 | nitrogen compound metabolic process | 5.83E-13 |
| macromolecule localization | 1.22E-15 | cellular nitrogen compound metabolic process | 6.60E-13 |
| nervous system development | 1.29E-15 | cellular component organization | 1.01E-12 |
| generation of neurons | 1.47E-15 | developmental process | 1.88E-12 |
| regulation of cellular metabolic process | 2.65E-15 | regulation of primary metabolic process | 2.32E-12 |
| neurogenesis | 5.31E-15 | biological regulation | 4.21E-12 |
| neuron differentiation | 6.33E-15 | gene expression | 5.85E-12 |
| regulation of metabolic process | 1.09E-14 | regulation of macromolecule metabolic process | 7.05E-12 |
| developmental process | 1.09E-14 | negative regulation of biological process | 1.81E-11 |
| cell development | 1.68E-14 | nucleobase, nucleotide and nucleic acid metabolism | 3.79E-11 |
| neuron development | 2.71E-14 | negative regulation of cellular process | 5.02E-11 |
| regulation of biosynthetic process | 5.86E-14 | regulation of gene expression | 6.03E-11 |
| cellular macromolecule metabolic process | 1.07E-13 | macromolecule biosynthetic process | 1.09E-10 |
| regulation of cellular biosynthetic process | 1.78E-13 | cellular macromolecule biosynthetic process | 1.36E-10 |
| cellular localization | 2.28E-13 | protein metabolic process | 1.64E-10 |
| regulation of primary metabolic process | 3.00E-13 | multicellular organismal development | 2.64E-10 |
| macromolecule metabolic process | 4.14E-13 | organelle organization | 9.11E-10 |
| macromolecule biosynthetic process | 4.33E-13 | regulation of nitrogen compound metabolism | 1.09E-09 |
| cell projection organization | 4.88E-13 | anatomical structure development | 1.14E-09 |
| multicellular organismal development | 8.95E-13 | regulation of cellular biosynthetic process | 1.17E-09 |
| regulation of nitrogen compound metabolism | 1.33E-12 | regulation of macromolecule biosynthetic process | 1.24E-09 |
| nucleobase, nucleotide and nucleic acid metabolism | 1.64E-12 | regulation of biological process | 1.74E-09 |
| nitrogen compound metabolic process | 2.04E-12 | regulation of biosynthetic process | 1.98E-09 |
| protein localization | 2.40E-12 | positive regulation of cellular process | 2.20E-09 |
| system development | 2.72E-12 | regulation of nucleic acid metabolism | 2.28E-09 |
| cellular developmental process | 2.99E-12 | protein modification process | 2.99E-09 |
| cell differentiation | 3.10E-12 | macromolecule modification | 3.03E-09 |
| cellular macromolecule biosynthetic process | 5.05E-12 | small molecule metabolic process | 6.39E-09 |
| regulation of macromolecule biosynthetic process | 9.65E-12 | nucleic acid metabolic process | 8.18E-09 |

Genomatix software was used to perform an unbiased analysis of biological processes that are associated with the genes at/near the CRX-overlapping or non CRX-overlapping NRL ChIP-Seq peaks. The top 40 biological processes in Illumina and ABI data are shown. **Overlap with CRX**: NRL ChIP-Seq peaks that overlap with CRX ChIP-Seq peaks. **Non-overlap with CRX**: NRL ChIP-Seq peaks that do not overlap with CRX ChIP-Seq peaks. Photoreceptor-related/specific biological processes are highlighted in green.
