# Supplementary material for: Transcriptional Regulation of Rod Photoreceptor Homeostasis Revealed by In Vivo NRL Targetome Analysis
Source: PLoS Genet. 2012 Apr 12;8(4):e1002649. doi: 10.1371/journal.pgen.1002649 (PMC3325202; doi:10.1371/journal.pgen.1002649)
Supplement: Table S5 — Identification of candidate retinal disease genes. Candidate retinal disease genes are based on the chromosomal location of the human orthologs of the NRL target genes that map within a mapped retinal disease locus reported in the RetNet database (www.sph.uth.tmc.edu/retnet/). The top part of the table lists the retinal disease genes that have been identified, whereas the bottom part lists the candidate genes in the region of uncloned but mapped disease locus. The fold enrichment value was produced by dividing the total number of genes (reported in RefSeq) within each locus by the number of NRL target genes at the same locus. (DOC) [file pgen.1002649.s010.doc]

**Table S5.** Identification of candidate retinal disease genes

| **Human orthologs of NRL target genes reported to cause retinal disease** | | | | | |
| --- | --- | --- | --- | --- | --- |
| **Known Disease gene** | **Location** | **Total NRL targets within the region** | **NRL targets within region** | **Total Genes within region** | **Fold enrichment** |
| AXPC1 | 1q31-q32 | 5 | Adipor1,Kdm5b,Zc3h11a,Klhl12,Nfasc | 200 | 40.0 |
| RP28 | 2p16-p11 | 1 | Fam161a | 238 | 238.0 |
| GNAT1 | 3p21.31 | 4 | Gnat1, Lrrc2, Usp4, Rbm5 | 144 | 36 |
| RHO, OPN2, RP4 | 3q22.1 | 1 | Rho | 47 | 47 |
| CNGA1, CNCG, CNCG1 | 4p12 | 2 | Cnga1, Fryl | 17 | 8.5 |
| CC2D2A | 4p15.33 | 2 | Cc2d2a, Rab28 | 8 | 4 |
| PDE6B, CSNB3, RP40 | 4p16.3 | 1 | Pde6b | 68 | 68 |
| BBS7, BBS2L1 | 4q27 | 1 | Bbs7 | 16 | 16 |
| GPR98, FEB4, MASS1, USH2C, VLGR1 | 5q14.3 | 2 | Gpr98, Mef2c | 18 | 9 |
| PDE6A | 5q33.1 | 2 | Pde6a, Sparc | 24 | 12 |
| GUCA1B, GCAP2 | 6p21.1 | 1 | Guca1b | 88 | 88 |
| RP1, ORP1 | 8q12.1 | 1 | Rp1 | 25 | 25 |
| RBP3, IRBP | 10q11.22 | 1 | Rbp3 | 28 | 28 |
| ROM1 | 11q12.3 | 2 | Rom1, Ttc9c | 61 | 30.5 |
| CABP4 | 11q13.1 | 2 | Cabp4, Cox8a | 110 | 55 |
| NRL, RP27 | 14q11.2 | 2 | Nrl, Rpgrip1 | 145 | 72.5 |
| RPGRIP1, LCA6 | 14q11.2 | 2 | Nrl, Rpgrip1 | 145 | 72.5 |
| RDH12 | 14q24.1 | 1 | Rdh12 | 24 | 24 |
| NR2E3, ESCS, PNR, RP37 | 15q23 | 1 | Nr2e3 | 49 | 49 |
| BBS2 | 16q12.2 | 1 | Bbs2 | 62 | 62 |
| PITPNM3, CORD5, NIR1 | 17p13.2 | 1 | Pitpnm3 | 70 | 70 |
| PGK1 | Xq21.1 | 1 | Pgk1 | 29 | 29 |

| **Human orthologs of NRL target genes within a mapped retinal disease locus**  **(for which the disease gene has not been identified)** |
| --- |

| **Disease** | **Location** | **Total NRL targets within the region** | **NRL targets within region** | **Total Genes within the region** | **Fold enrichment** |
| --- | --- | --- | --- | --- | --- |
| RP32 | 1p21.2-p13.3 | 11 | Gadd45a,Spata1,Eif2c3,Gpsm2,Extl2,Usp33,Ahcyl1,St3gal3,Negr1,Leprot,Hs2st1 | 555 | 50.5 |
| LCA9 | 1p36 | 10 | Gnb1,Dhrs3,Rnf207,Kif1b,Clstn1,Ece1,Clic4,Alpl,Tmem201,Usp48 | 415 | 41.5 |
| CORD8 | 1q23.1-q23.3 | 1 | Cadm3 | 131 | 131.0 |
| RP33 | 2cen-q12.1 | 2 | Stard7,Rpl31 | 90 | 45.0 |
| LOC619531 | 2q11 | 2 | Stard7,Rpl31 | 80 | 40.0 |
| CRV, HERNS, HVR | 3p21.3-p21.1 | 4 | Lrrc2,Usp4,Rbm5,Vprbp | 213 | 53.3 |
| USH2B | 3p24.2-p23 | 3 | Slc4a7,Azi2,Ube2e1 | 23 | 7.7 |
| WFS2 | 4q22-q24 | 1 | Herc3 | 81 | 81.0 |
| RP29 | 4q32-q34 | 3 | Galnt7,Gria2,Sap30 | 81 | 27.0 |
| MCDR3 | 5p15.33-p13.1 | 3 | Egflam,Pdzd2,Lifr | 106 | 35.3 |
| BSMD | 5q21.2-q33.2 | 4 | Ppargc1b,Trim36,Pcdhac2,Sap30l | 369 | 92.3 |
| BCMAD | 6p12.3-q16 | 5 | Pla2g7,Me1,Bach2,Nt5e,Phip | 195 | 39.0 |
| MCDR1, NCMD, PBCRA1 | 6q14-q16.2 | 4 | Me1,Bach2,Nt5e,Phip | 87 | 21.8 |
| RCD1 | 6q25-q26 | 3 | Zdhhc14,Syne1,Lrp11 | 81 | 27.0 |
| MDDC, CYMD | 7p21-p15 | 3 | Ccdc126,Ahr,Arl4a | 93 | 31.0 |
| OPA6, ROA1 | 8q21-q22 | 5 | Plekhf2,Terf1,Tceb1,Pabpc1,Sdc2 | 141 | 28.2 |
| CORS2, JBTS2 | 11p12-q13.3 | 5 | Cox8a,Ttc9c,Vps37c,Harbi1,Mrpl11 | 498 | 99.6 |
| EVR3 | 11p13-p12 | 2 | Slc1a2,Cstf3 | 45 | 22.5 |
| VRNI | 11q13 | 3 | Cox8a,Mrpl48,Mrpl11 | 271 | 90.3 |
| CODA1 | 12q13.13-q14.3 | 1 | Dyrk2 | 213 | 213.0 |
| MRST | 15q24 | 1 | Scaper | 76 | 76.0 |
| RP22 | 16p12.3-p12.1 | 2 | Cdr2,Mettl9 | 90 | 45.0 |
| CACD | 17p13 | 2 | Gas7,Rtn4rl1 | 254 | 127.0 |
| CORD4 | 17q | 14 | Slc16a6,Cuedc1,Plcd3,Tex2,Dgke,Mmd,Nup85,Nsf,Prkca,Acly,Slc38a10,Npepps,Rpl27,Ssh2 | 853 | 60.9 |
| OPA4 | 18q12.2-q12.3 | 1 | Haus1 | 26 | 26.0 |
| CORD1 | 18q21.1-q21.3 | 2 | Nedd4l,Lman1 | 80 | 40.0 |
| OPA5 | 22q12.1-q13.1 | 3 | Thoc5,Nhp2l1,Tef | 222 | 74.0 |
| RP with mental retardation | Xp21-q21 | 2 | Arr3,Ogt | 372 | 186.0 |
| RP23 | Xp22 | 1 | Gpm6b | 129 | 129.0 |

Candidate retinal disease genes are based on the chromosomal location of the human orthologs of the NRL target genes that map within a mapped retinal disease locus reported in the RetNet database (www.sph.uth.tmc.edu/retnet/). The top part of the table lists the retinal disease genes that have been identified, whereas the bottom part lists the candidate genes in the region of uncloned but mapped disease locus. The fold enrichment value was produced by dividing the total number of genes (reported in RefSeq) within each locus by the number of NRL target genes at the same locus.
