# Supplementary material for: Transcriptional Regulation of Rod Photoreceptor Homeostasis Revealed by In Vivo NRL Targetome Analysis
Source: PLoS Genet. 2012 Apr 12;8(4):e1002649. doi: 10.1371/journal.pgen.1002649 (PMC3325202; doi:10.1371/journal.pgen.1002649)
Supplement: Text S1 — Supplemental extended experimental procedures. (DOC) [file pgen.1002649.s011.doc]

**Transcriptional Regulation of Rod Photoreceptor Homeostasis Revealed by In Vivo NRL Targetome Analysis**

Hong Hao, Douglas S. Kim, Bernward Klocke, Kory Johnson, Kairong Cui, Norimoto Gotoh, Chongzhi Zang, Janina Gregorski, Linn Gieser, Weiqun Peng, Yang Fann, Martin Seifert, Keji Zhao, Anand Swaroop

**Supplemental Extended Experimental Procedures**

**Animals**

C57Bl/6J and CD1 mice were purchased from Jackson Laboratory and Charles River Laboratories, respectively. Animal Care and Use Committee of the National Eye Institute approved all mouse protocols.

**ChIP-Seq by Illumina and ABI/SOLiD platforms**

Retina from postnatal day (P) 28 mice was used for ChIP experiments with NRL antibody or normal IgG, as previously described (1). Briefly, retinas were dissected in 1 x phosphate-buffered saline (PBS), crosslinked with 1% formaldehyde for 15 min at room temperature. The crosslinking was stopped by incubation with 0.125 M glycine for 5 min. Cells were lysed in Lysis buffer (50 mM HEPES-KOH, pH 8, 1 mM EDTA, 0.5 mM EGTA, 140 mM NaCl, 10% glycerol, 0.5% nonidet P-40 0.25%, Triton X-100). The nuclear pellet was suspended in RIPA buffer (10 mM Tris-HCl, pH 8,1 mM EDTA, 0.5 mM EGTA, 140 mM NaCl, 1% Triton X-100, 0.1% Na-deoxycholate, 0.1% SDS) and sonicated for 10 cycles of 30s of sonication (Misonix sonicators) followed by 30s resting intervals. Samples were centrifuged for 10 min at 18,000 g and the supernatant was split in half for immunoprecipitation with NRL antibody or normal IgG (Upstate) at 4^o^C overnight. After incubation with 50 ul of Protein A beads (Invitrogen 100-04D) for 6 h, the samples were washed, incubated at 65 ^o^C overnight, treated with RNase and proteinase K. The ChIP DNA was purified by Phenol/Chloroform extraction and checked for enrichment of *Rhodopsin* promoter by qPCR, then the ChIP DNA from multiple sets of ChIP assays were pooled for preparation of ChIP-Seq libraries. Fifteen or 25 ng of ChIP DNA from independent experiments was used for library preparations following manufacturers’ instructions and sequenced on Illumina 1G Genome Analyzer or ABI SOLiD V2 system, respectively.

**Mapping of ChIP-Seq reads**

Raw sequencing reads from Illumina or ABI platforms were mapped to the mouse genome (NCBI build 37) using Genomatix Mining Station (GMS). A minimal 92% nucleotide identity to the reference genome was required for mapping to the genome with no insertions or deletions. Reads that uniquely mapped to the genome were used for downstream analyses.

**NRL ChIP-Seq peak calling**

ChIP-Seq peaks were called using MACS (2) at p<10^-5^ and NGS-Analyzer (Genomatix) at p<10^-3^ value. Both programs call peaks according to a Poisson distribution model. NGS-Analyzer calculates the threshold for clustering from the ChIP reads (3), and a window size of 100 bp was used for clustering. Read counts for ChIP peaks from the NRL antibody library were compared to the read counts in the same genomic regions from the IgG control library (4). The union of overlapping peak regions from both methods was used in subsequent analyses.

**Comparison of NRL ChIP-Seq peaks with CRX ChIP-Seq data**

The NRL ChIP-Seq peaks were compared to the CRX ChIP-Seq regions (5) using GenomeInspector (Genomatix; (6)). Each region spanning -2000 bp to +2000 bp from the CRX peak center was scanned for an overlap with NRL ChIP-Seq peaks. The CRX peak centers were aligned as position 0, and the number of overlaps of NRL ChIP-Seq peaks was shown as a correlation plot. NRL ChIP-Seq peaks that overlap within 500 bp of CRX peak centers were presented in the Venn diagram.

**Transcription factor enrichment analysis**

Transcription factor binding site enrichment analyses of sequences in ChIP-Seq peak regions were performed using RegionMiner and MatInspector (Genomatix; (6,7)). The ChIP-Seq peak regions were scanned for matches to the matrices of vertebrate transcription factor family binding site (TFBS) in Genomatix MatBase version 8.2 (7). The results were compared to the pre-compiled matches in the whole mouse genome and all annotated mouse promoters (Genomatix ElDorado database). The Z-score of overrepresentation against the selected background is the distance from the population mean in units of the population standard deviation. Here, Z-scores are calculated with a continuity correction using the formula z = (x-E-0.5)/S, where x is the number of found matches in the input data, E is the expected value and S is the standard deviation.

**Calculation of the TFBS positional-bias**

The positional bias was calculated as outlined for the assessment of *de novo* detected motives (8). The ChIP-Seq peaks were extended to -500 bp and +500 bp from the peak center. The sequences were scanned for TFBS matrices (Genomatix MatBase version 8.2) using MatInspector (Genomatix). For positional bias calculation (8), overlapping 100 bp sliding windows were used in steps of 10 bp. The total number of matches for a given TFBS family is regarded as independent individual trials that may hit anywhere in the sequence. The positional bias is calculated as the cumulative binomial probability of finding certain number of matches up to the total number of matches in a scan window. The probability of observing a single match within a scan window, independent of sequence constraints, is given as the fraction of window/sequence length. The positional bias (P) was calculated for each matrix family and each scan window using a customized perl script. For graphical visualization, the –log(P) was plotted against the scan windows’ mid-positions using the R software environment (http://www.r-project.org/). The over-represented TFBS positions for a TF family appear as peaks in these plots.

**Gene expression analysis**

Previously generated Affymetrix microarray data from flow-sorted photoreceptors of WT and *Nrl*^-/-^ retina (9) was analyzed using ChipInsepector (Genomatix). The statistic algorithm in ChipInspector is a t-test with a permuted artificial background (10). Cut-off values for the measured probe values deviant from expectation were set to yield an estimated False Discovery Rate (FDR) of 0%.

**ChIP-quantitative PCR (qPCR)**

Independent ChIP experiments were performed to validate ChIP-Seq data by qPCR. ChIP DNA from 3 IP experiments was pooled and then tested in triplicates by qPCR for enrichment of target regions. The complete procedure from ChIP to qPCR was repeated once. For comparison of NRL antibody and IgG using WT retinas, total 120 WT retinas were used for 2 sets of 3 NRL ChIP and 3 IgG ChIP using 10 retinas for each ChIP. For the comparison of WT retinas and *Nrl*^-/-^ retinas using NRL antibody, total 60 WT retinas and 60 *Nrl*^-/-^ retinas were used in 2 sets of 3 IP using WT retinas and 3 IP using *Nrl*^-/-^ retinas with 10 retinas for each IP.

ChIP DNA was tested in triplicates for the enrichment of randomly selected ChIP-Seq regions by qPCR using SYBR Green (11). To correlate the sequence tag numbers with the fold change detected by qPCR, we randomly picked 26 regions with peaks covering the majority range of the peak heights. Six regions without ChIP-Seq signals served as negative controls. Normal rabbit IgG served as the negative antibody control, and *Nrl*^-/-^ retina was used as a negative tissue control.

**Cell Culture, transfection, plasmids and cloning**

HEK293T cells were cultured in DMEM supplemented with 10% FBS at 37^o^C with 5% CO_2_, and transfected with Fugene 6 (Roche) according to manufacturer’s instruction. The shRNA plasmids and cDNA expression plasmids for the NRL target genes (tested in this study), and the shRNA plasmid targeting *Gapdh* (the negative control) were purchased from Open Biosystems. To generate shRNA-resistant dcDNA constructs, the cDNAs of *Lman1* and *Wisp1* were first sub-cloned to a vector under the control of ubiquitin C (Ub) promoter. Silent mutations that confer resistance to shRNA were introduced using Quikchange kit (Stratagene). shRNA target sequence and primer information are provided on the web site www.nei.nih.gov/intramural/nnrldataresource.asp .

**Enhancer analysis using luciferase assays**

To functionally evaluate ChIP-Seq peak sequences for possible enhancer activity, the peak regions (~ 500 bp) were amplified from genomic DNA and cloned into KpnI and NheI sites or MluI and XhoI sites of pGL3-promoter vector (SV40 minimal promoter driving firefly luciferase; Promega) and verified by sequencing. HEK293T cells were transfected with enhancer constructs, an internal control plasmid expressing Renilla luciferase (Promega), and NRL expression plasmid or empty vector pC4C. The luciferase activities of the cell lysate were measured 48 hr after transfection using Dual-luciferase Reporter Assay System (Promega).

**shRNA-sensor assay**

To test the efficiency of the shRNA plasmids, we generated shRNA-sensor constructs by cloning the shRNA target sequences (as oligonucleotides) into the 3’UTR of a GFP vector. The shRNA-sensor construct and CAG-HcRed as a transfection control were co-transfected with either shRNA to target protein or control shRNA to *Gapdh*, and assayed 48 h after transfection. Decrease in GFP fluorescence compared to controls indicated the effective knockdown by a given shRNA construct (Figure S1). For each target gene, three different shRNA constructs were evaluated for efficacy, and the most efficient one was chosen for *in vivo* knockdown experiments.

**Sub-retinal injection and *in vivo* electroporation**

shRNA alone or together with shRNA-resistant dcDNA was introduced in the retina of CD-1 P0 mouse pups by sub-retinal injection followed by *in vivo* electroporation, as previously described (12,13). The injection volume was 150 nL, and DNA concentration for each plasmid was 600 nM. The retinas were harvested at postnatal day 7 or 20, and sectioned for histology or immunohistochemistry.

**Immunohistochemistry**

Retina was dissected, fixed in 4% paraformaldehyde, and cryoprotected in 30% sucrose. Slide-mounted, 10-µM sections were stained with a mouse monoclonal anti-Rhodopsin antibody (Rho4D2, 1:100; Robert Molday, University of British Columbia) and an Alexa Fluor 568 goat anti-mouse IgG secondary antibody (1:300; Invitrogen, Carlsbad, CA) and then counterstained with DAPI (1 µg/mL). Photomicrographs were obtained using an Olympus FluoView FV1000 confocal laser scanner and BX61WI microscope (Center Valley, PA).

**Retina dissociation, FACS isolation, and exon arrays**

Mouse retina was electroporated at P0 with Ub-GFP and *Gapdh* shRNA or *Kdm5b* shRNA and dissected at P20. GFP+ retinal cells were isolated from dissociated retina by FACS (FACSAria; BD Biosciences). RNA was extracted from 1–5 × 10*^5^* flow-sorted cells, and cDNA was synthesized using Ovation Pico WTA System, followed by sense transcript cDNA (ST-cDNA) generation using WT-Ovation Exon module as described by the manufacturer (NuGEN Technologies, San Carlos, CA). The ST-cDNA was fragmented and labeled with Encore Biotin Module (NuGen). Then, 5 μg of biotinylated ST-cDNA was incubated with GeneChip® Mouse Exon 1.0 ST array (Affymetrix) at 45°C for 18–20 hours. Arrays were washed and stained using the Hybridization, Wash and Stain kit according to manufacturer’s instructions (Affymetrix) and subsequently scanned. The microarray data has been deposited in the Gene Expression Omnibus Database (accession #: will be available soon).

**Exon array data analysis**

Raw exon array data was extracted to the CEL files with Affymetrix Power Tools software package (Affymetrix). Partek Genomic Suite Software was used to perform the probe-set signal estimation and yielded average probe-set intensity value in each group (Partek, St. Louis). Full probe-set list was subjected to gene level intensity estimation. AltAnalyze was used with a cut-off parameter of p < 0.01 by the Detection Above BackGround (DABG) statistics (14). The log 2 intensity value of 5 was adopted for the minimum acceptable averaged signal intensity level in each group**.**

**Candidate gene extraction with filtering**

The criteria for differentially expressed genes included: (1) Fold change calculation in every probe-set by using the average intensity values when comparing between *Kdm5b* suppression *versus* control group, as described (15). For each transcript cluster, we counted the number of probe-sets with more than 2-fold change. An orientation filter (number of up probe-set/number of down probe-set ≥ 5, ≤ 1/5, or 0 probe-set) was applied to deal with the expression change in both directions. (2) Genes represented with less than 5 probe-sets were subjected to fold change criterion only. Such genes include many RIKEN genes and putative transcripts. The genes with less annotation in public databases were discarded from analysis. (3) After applying the preceding criteria, we eliminated genes showing less than 1.5 fold change based on gene-level signal intensity. (4) We also removed genes that showed p>0.1 after unpaired t-test for the two groups with three biological replicates.

We performed a GO analysis and gene pathway analysis of significant genes with the Ingenuity Pathways Analysis (IPA) software, version 6.0 (Ingenuity Systems, Mountain View, CA). The genes with a minimal 2-fold change in *Kdm5b* shRNA were compared to the list of genes with 1.5 fold change between photoreceptors of WT or *Nrl*^-/-^ mice (9).

**References**

1. Oh, E. C., Cheng, H., Hao, H., Jia, L., Khan, N. W., and Swaroop, A. (2008) *Brain Res* 1236, 16-29

2. Zhang, Y., Liu, T., Meyer, C. A., Eeckhoute, J., Johnson, D. S., Bernstein, B. E., Nussbaum, C., Myers, R. M., Brown, M., Li, W., and Liu, X. S. (2008) *Genome Biol* 9, R137

3. Sultan, M., Schulz, M. H., Richard, H., Magen, A., Klingenhoff, A., Scherf, M., Seifert, M., Borodina, T., Soldatov, A., Parkhomchuk, D., Schmidt, D., O'Keeffe, S., Haas, S., Vingron, M., Lehrach, H., and Yaspo, M. L. (2008) *Science* 321, 956-960

4. Audic, S., and Claverie, J. M. (1997) *Genome Res* 7, 986-995

5. Corbo, J. C., Lawrence, K. A., Karlstetter, M., Myers, C. A., Abdelaziz, M., Dirkes, W., Weigelt, K., Seifert, M., Benes, V., Fritsche, L. G., Weber, B. H., and Langmann, T. (2010) *Genome Res*

6. Quandt, K., Frech, K., Karas, H., Wingender, E., and Werner, T. (1995) *Nucleic Acids Res* 23, 4878-4884

7. Cartharius, K., Frech, K., Grote, K., Klocke, B., Haltmeier, M., Klingenhoff, A., Frisch, M., Bayerlein, M., and Werner, T. (2005) *Bioinformatics* 21, 2933-2942

8. Hughes, J. D., Estep, P. W., Tavazoie, S., and Church, G. M. (2000) *J Mol Biol* 296, 1205-1214

9. Akimoto, M., Cheng, H., Zhu, D., Brzezinski, J. A., Khanna, R., Filippova, E., Oh, E. C., Jing, Y., Linares, J. L., Brooks, M., Zareparsi, S., Mears, A. J., Hero, A., Glaser, T., and Swaroop, A. (2006) *Proc Natl Acad Sci U S A* 103, 3890-3895

10. Tusher, V. G., Tibshirani, R., and Chu, G. (2001) *Proc Natl Acad Sci U S A* 98, 5116-5121

11. Zipper, H., Brunner, H., Bernhagen, J., and Vitzthum, F. (2004) *Nucleic acids research* 32, e103

12. Matsuda, T., and Cepko, C. L. (2004) *Proc Natl Acad Sci U S A* 101, 16-22

13. Kautzmann, M. A., Kim, D. S., Felder-Schmittbuhl, M. P., and Swaroop, A. (2011) *J Biol Chem*

14. Emig, D., Salomonis, N., Baumbach, J., Lengauer, T., Conklin, B. R., and Albrecht, M. (2010) *Nucleic acids research* 38, W755-762

15. Bradford, J. R., Hey, Y., Yates, T., Li, Y., Pepper, S. D., and Miller, C. J. (2010) *BMC genomics* 11, 282.
